# No evidence for magnetic field effects on the behaviour of *Drosophila*

**DOI:** 10.1038/s41586-023-06397-7

**Published:** 2023-08-09

**Authors:** Marco Bassetto, Thomas Reichl, Dmitry Kobylkov, Daniel R. Kattnig, Michael Winklhofer, P. J. Hore, Henrik Mouritsen

**Affiliations:** 1 Physical & Theoretical Chemistry Laboratory, Department of Chemistry, University of Oxford, Oxford, UK; 2 AG Neurosensory Sciences/Animal Navigation, Institut für Biologie und Umweltwissenschaften, Carl-von‐Ossietzky Universität Oldenburg, Oldenburg, Germany; 3 Center for Mind/Brain Science, University of Trento, Rovereto, Italy; 4 Living Systems Institute, University of Exeter, Exeter, UK; 5 Department of Physics, University of Exeter, Exeter, UK; 6 AG Sensory Biology of Animals, Institut für Biologie und Umweltwissenschaften, Carl-von‐Ossietzky Universität Oldenburg, Oldenburg, Germany; 7 Research Center for Neurosensory Sciences, University of Oldenburg, Oldenburg, Germany

**Keywords:** Biological models, Motivation

## Abstract

Migratory songbirds have the remarkable ability to extract directional information from the Earth’s magnetic field^1,2^. The exact mechanism of this light-dependent magnetic compass sense, however, is not fully understood. The most promising hypothesis focuses on the quantum spin dynamics of transient radical pairs formed in cryptochrome proteins in the retina^3–5^. Frustratingly, much of the supporting evidence for this theory is circumstantial, largely because of the extreme challenges posed by genetic modification of wild birds. *Drosophila* has therefore been recruited as a model organism, and several influential reports of cryptochrome-mediated magnetic field effects on fly behaviour have been widely interpreted as support for a radical pair-based mechanism in birds^6–23^. Here we report the results of an extensive study testing magnetic field effects on 97,658 flies moving in a two-arm maze and on 10,960 flies performing the spontaneous escape behaviour known as negative geotaxis. Under meticulously controlled conditions and with vast sample sizes, we have been unable to find evidence for magnetically sensitive behaviour in *Drosophila*. Moreover, after reassessment of the statistical approaches and sample sizes used in the studies that we tried to replicate, we suggest that many—if not all—of the original results were false positives. Our findings therefore cast considerable doubt on the existence of magnetic sensing in *Drosophila* and thus strongly suggest that night-migratory songbirds remain the organism of choice for elucidating the mechanism of light-dependent magnetoreception.

## Abstract (web summary)

Following testing of magnetic field effects on 97,658 flies moving in a two-arm maze and on 10,960 flies performing spontaneous escape behaviour (negative geotaxis), no evidence was found for magnetically sensitive behaviour in *Drosophila.*

## Main

Most of our knowledge of light-dependent magnetoreception originates from night-migratory songbirds, which show highly reproducible compass responses when tested during the migratory season in orientation cages such as Emlen funnels^24–26^ and in free flight^27^. They also seem to combine their homeward compass bearing with a magnetic inclination-based ‘stop sign’ to decide where to end their return journey^28^. Working with such birds is challenging because they cannot routinely be bred in captivity and many modern genetic approaches are inapplicable. We were therefore interested to see reports that *Drosophila* show magnetically influenced behaviours^6–23^. Even though the evolutionary benefit of exploiting magnetic cues is unclear, a broadly reproducible behavioural paradigm to test for magnetoreception in *Drosophila* would greatly facilitate the search for the exact mechanisms of, sensory molecules for, genetic basis of and neuronal responses to magnetic stimuli. It would be much more difficult to achieve the same level of knowledge and insight using only night-migratory songbirds. We therefore decided to implement two of the published *Drosophila* behavioural assays in our own laboratories.

**Extended Data Fig. 1:**
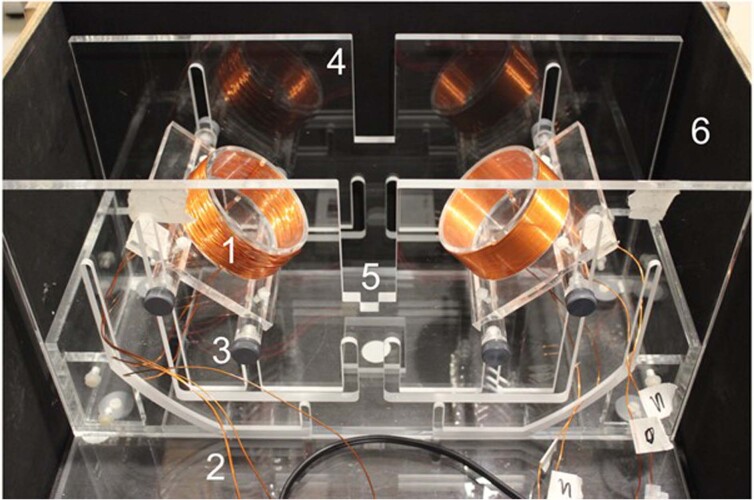
Photograph of the T-maze apparatus. **1**. Double-wound coils. **2**.Cables to and from the coils. **3**. Screw keeping the coils in position. **4**. Notch that allowed the T-maze elevator to be kept in the horizontal position during the testing phase. **5**. The lower rectangular notch allowed the T-maze elevator to be kept in the vertical position during the training phase. **6**. Black wooden box.

We first tried the binary-choice, T-shaped maze assay of Gegear et al.^6,7^ and Foley et al.^8^ with an exact replica of the original apparatus and following the published protocols and additional information provided by the original authors (Extended Data Fig. 1). A magnetic field of around 500 µT was applied in one arm of the maze and no magnetic field in the other, by passing identical currents parallel and antiparallel, respectively, through identical double-wrapped coils. This arrangement ensures that any non-magnetic effects, such as minor heating, would be the same in the two arms. The apparatus, together with white striplights, was contained within a wooden box placed inside an electromagnetically shielded chamber (4.0 × 5.0 × 2.5 m^3^) in a wooden building that attenuated background radiofrequency fields by a factor of at least 10^5^ (ref. ^29^). In this way flies, tested in groups of approximately 100, were exposed to the static field produced by the coils and/or to the Earth’s magnetic field but not to radiofrequency electromagnetic fields, which have been found to interfere with birds’ ability to use their magnetic compass^26,29,30^.

**Fig. 1:**
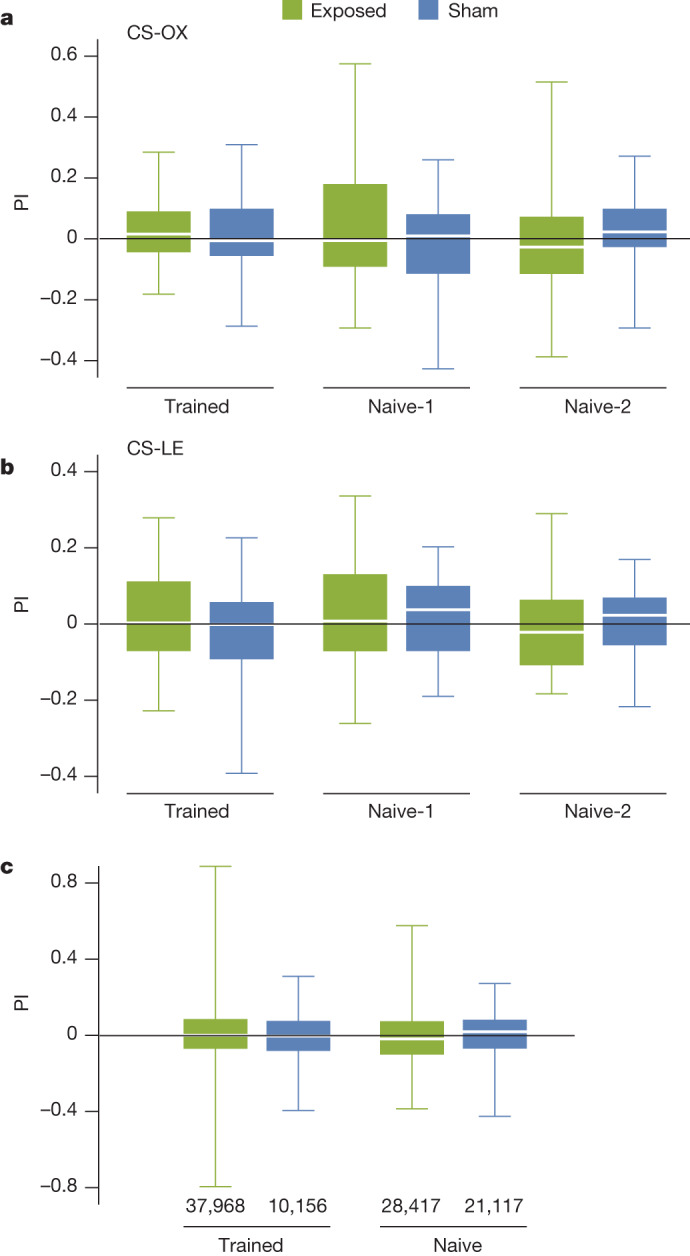
Testing of *Drosophila* magnetic sensing in the T-maze assay. **a**–**c**, Preference index, defined as ${\rm{PI}}=2{P}_{{\rm{M}}}-1$ where *P*_M_ is the proportion of flies that preferred the magnetic arm of the maze. Flies were tested with (exposed) and without (sham) a magnetic field of approximately 500 µT in one arm of the maze. The trained and ‘naive-1’ data were obtained using the exact protocols of Gegear et al.^6^. The ‘naive-2’ data were obtained from flies tested under the same conditions as the trained flies except for omission of the sucrose reward (Supplementary Information). **a**, CS-OX flies. **b**, CS-LE flies. **c**, Pooled data for all flies tested in the T-maze, including all experiments described in Supplementary Information. Total numbers of flies tested are shown. For each condition in **a** and **b**, 50 independent sets, all of around 100 flies, were tested in each experimental condition. In no case did flies show even a marginally significant (*P* < 0.1) preference for, or avoidance of, the magnetic field (Supplementary Information). Box plots show the median, 25 and 75% quartiles and maximum and minimum values.

**Extended Data Fig. 2:**
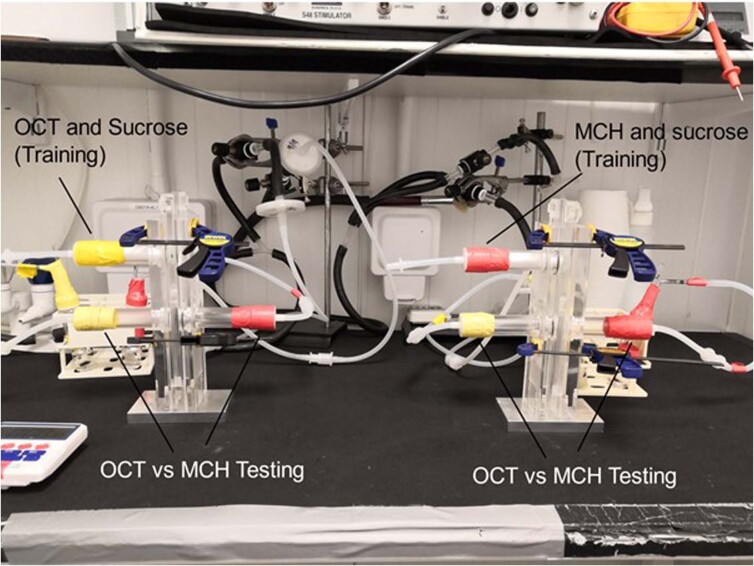
Photograph of apparatus for odour conditioning control experiments. Equipment used to test flies’ ability to associate an odour with the sucrose reward used in the magnetic conditioning experiments. In the left-hand T-maze, flies were trained to associate OCT with sucrose (with MCH as US). Vice-versa in the right-hand T-maze.

In the original studies^6–8^, ‘naive’ flies that had not previously experienced the 500 µT magnetic field were reported to avoid this field in the maze whereas ‘trained’ flies, which had been conditioned to associate a sucrose reward with a single exposure to a 500 µT field, preferred the magnetic arm of the maze. We tested the strain of wild-type Canton-S flies (CS-OX) for which the strongest magnetic responses had been reported by Gegear et al.^6,7^ and Foley et al.^8^, and another Canton-S strain (CS-LE) from a different laboratory, all under the same conditions as the original studies. In both cases we saw no preference for, or avoidance of, the 500 μT magnetic field in either naive or trained flies (Fig. 1a,b). By contrast, using similar procedures and sucrose rewards, our flies were readily conditioned to odours (Supplementary Fig. 4, Supplementary Table 5 and Extended Data Fig. 2).

Having been unable to replicate the original findings, we searched for alternative experimental conditions under which *Drosophila* might show either a spontaneous magnetic preference or a magnetic field-conditioned response in the T-maze (Supplementary Methods). For instance, given the scarcity of reports of animals able to associate a magnetic field with a reward after a single exposure^31^, we increased the number of training sessions from one to four. We also tested wild-caught *Drosophila*. In both cases we found no magnetic field effect (Supplementary Figs. 2 and 3 and Supplementary Tables 2 and 3). Other variations in the experimental protocol (different sucrose concentrations, flies of different ages, flies reared under natural light and flies tested without electromagnetic shielding (Supplementary Figs. 2 and 3 and Supplementary Tables 2 and 3)) also failed to elicit a magnetic preference. In conclusion, after testing 984 sets of around 100 flies each (97,658 flies in total) over a period of 48 months, we found neither preference for nor avoidance of the magnetic field in the T-maze for either trained or naive flies (Fig. 1c).

In contrast to the original T-maze experiments^6–8^, our tests were performed in a completely non-magnetic research facility in which both static and time-dependent magnetic fields were meticulously controlled^29^. We also used a much larger number of independent replicates. Furthermore, our experimenters were blind to magnetic conditions in all experiments.

Noting the large scatter about the zero-preference value in most of our magnetic field experiments, we wondered whether the magnetic field effects in the original work could reflect statistical fluctuations, which are known to produce false positives and are likely to occur for small sample sizes (the ‘winner’s curse’ phenomenon^32^). We therefore reassessed the statistical analysis in Gegear et al.^6^ (Supplementary Fig. 1 and Supplementary Table 1) and realized that the authors had analysed their data by implicitly assuming that each individual fly acted as a true biological replicate, making its choice independently of the other (roughly) 100 flies in the maze. The assumption of independence is crucial for the applicability of the *t*-test to preference data but is violated in the group assay, which leads to inflation of significance by pseudoreplication. By applying the *t*-test, the authors thus obtained very highly significant *P* values for fairly small differences between the proportions of flies that avoided or preferred the magnetic arm of the maze. When we used a statistical framework suitable for proportion data, we furthermore found that, even for the largest apparent magnetic field effect reported for this assay^6–8^, as presented in Fig. 1b of Gegear et al.^6^ (44.5% naive versus 58.5% trained), the statistical power achieved was only about 10% with 10 and 12 sets of flies in each group. This means that, with such small sample sizes, the null hypothesis of no magnetic field effect could not be rejected in more than 90% of such cases. From these considerations, in combination with our own results, we conclude that the originally reported magnetic field effects were false positives (Supplementary Table 4).

Having been unsuccessful in reproducing the reported T-maze results^6–8^, we turned to a spontaneous *Drosophila* behaviour, with no requirement for conditioning, for which magnetic field effects had also been reported. In an investigation of the innate escape response known as negative geotaxis, Fedele et al.^9^ tested groups of ten flies inside plastic tubes placed between double-wrapped coils under either ‘exposed’ (roughly 500 µT applied field) or ‘sham’ (no applied field) conditions. Under dim blue light, the magnetic field was found to reduce the rate at which the flies climbed after being knocked down to the bottom of a tube^9^.

**Fig. 2:**
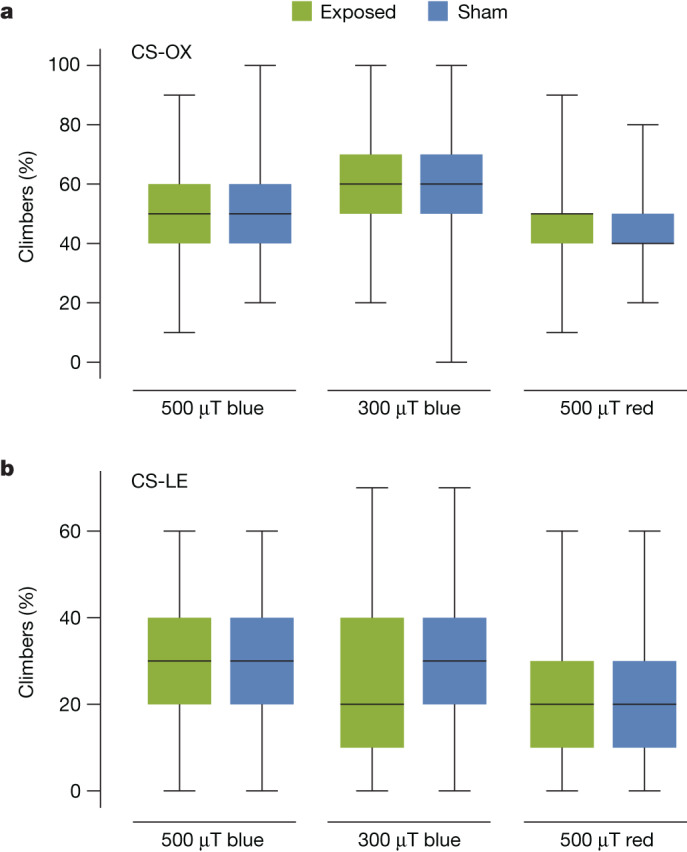
Testing of *Drosophila* magnetic sensing in the original negative-geotaxis assay. **a**,**b**, Percentages of flies (CS-OX, **a**; CS-LE, **b**) that climbed 15 cm in 15 s. In total, 150 sets of ten flies were tested in each experimental condition. No statistically significant differences in climbing behaviour were found for either 500 or 300 µT magnetic field exposures compared with sham exposure conditions with no applied magnetic field. Flies tested under blue light climbed significantly faster than those tested under red light (Supplementary Information). Box plots show the median, 25 and 75% quartiles and maximum and minimum values.

**Extended Data Fig. 3:**
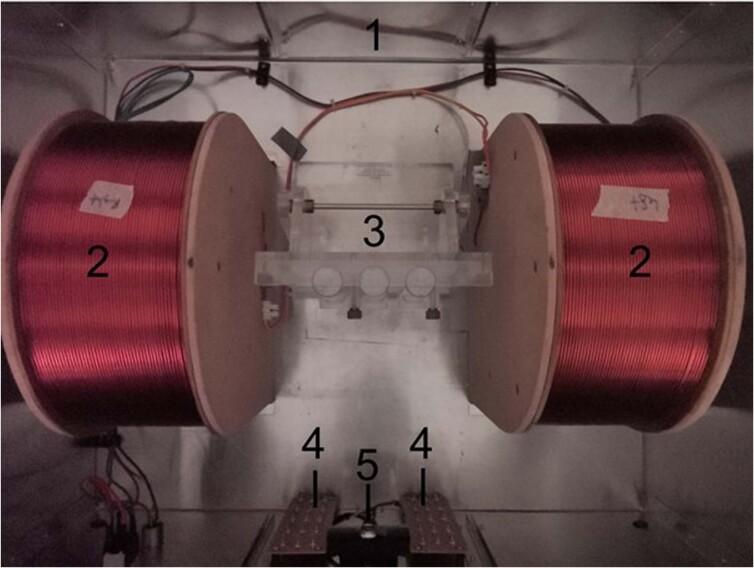
Photograph of the original negative geotaxis apparatus. **1**. Aluminium box. **2**. Double-wrapped coils. **3**. Swinger apparatus. **4**. LEDs strips. **5**. Infrared camera. This same setup was used both in ref. ^9^ and by us.

To replicate Fedele et al.^9^ we tested the same *Drosophila* strain (CS-LE) and used the apparatus built for the original study (Extended Data Fig. 3). The measurements were performed inside the electromagnetically screened chambers described above^29^ by experimenters who were blind to the magnetic field conditions. The flies were filmed and, as in the original study^9^, the proportion that climbed 15 cm in 15 s was recorded. We found no difference between flies tested under dim blue light with and without a 500 µT applied magnetic field (Fig. 2). Similar results were obtained using a 300 µT field and when the tests were repeated for another *Drosophila* strain (CS-OX; Fig. 2). Although no magnetic field effects were detected, we were able to replicate the positive-control observation of Fedele et al.^9^ that flies exposed to red light climb less rapidly—independent of the magnetic field—than those tested under blue light (Fig. 2).

**Fig. 3:**
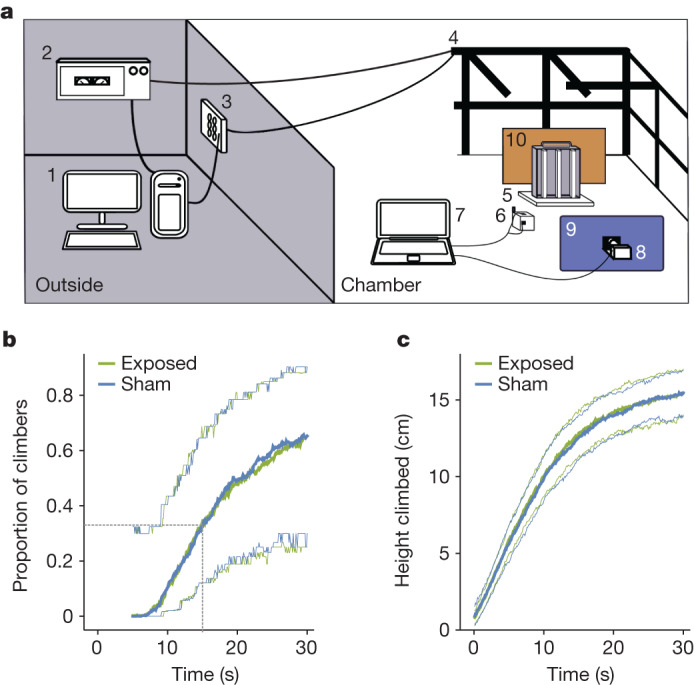
Testing of *Drosophila* magnetic sensing in the gravity negative-geotaxis assay. **a**, Schematic of the apparatus. A desktop computer (1), situated outside the shielded chamber, controlled the power supplies (2), the switching box (3) and the double-wrapped three-dimensional Merritt four-coil system (4). Large parts of the 12 coils have been omitted for visual clarity; a photograph of the chamber and coils can be found in ref. ^29^. Tubes containing the flies (5) were placed in the centre of the coils. Temperature, light and magnetic field sensors (6) monitored the experiments continuously. These sensors were connected to a laptop (7) that also controlled the video camera (8). The flies were illuminated with UV-blue, blue or red light via an array of LEDs (9). A second LED array (10) generated infrared light for filming. **b**, Climbing behaviour of CS-LE flies under blue light (410–490 nm), showing the time dependence of the proportion of flies reaching 15 cm when exposed to a 300 µT magnetic field compared with sham exposure conditions with no applied magnetic field. Data are shown as mean (thick lines) and mean ± confidence interval (thin lines) for proportion data. Grey dashed line indicates the proportion of flies able to climb 15 cm in 15 s. **c**, Average height climbed by flies as a function of time (same conditions as **b**). Data are shown as mean (thick lines) and mean ± s.d. (thin lines). No significant difference between sham (0 μT) and exposed (300 µT) conditions was found. **b**,**c**, Data for 15 groups of ten flies in each condition (exposed and sham).

**Extended Data Fig. 4:**
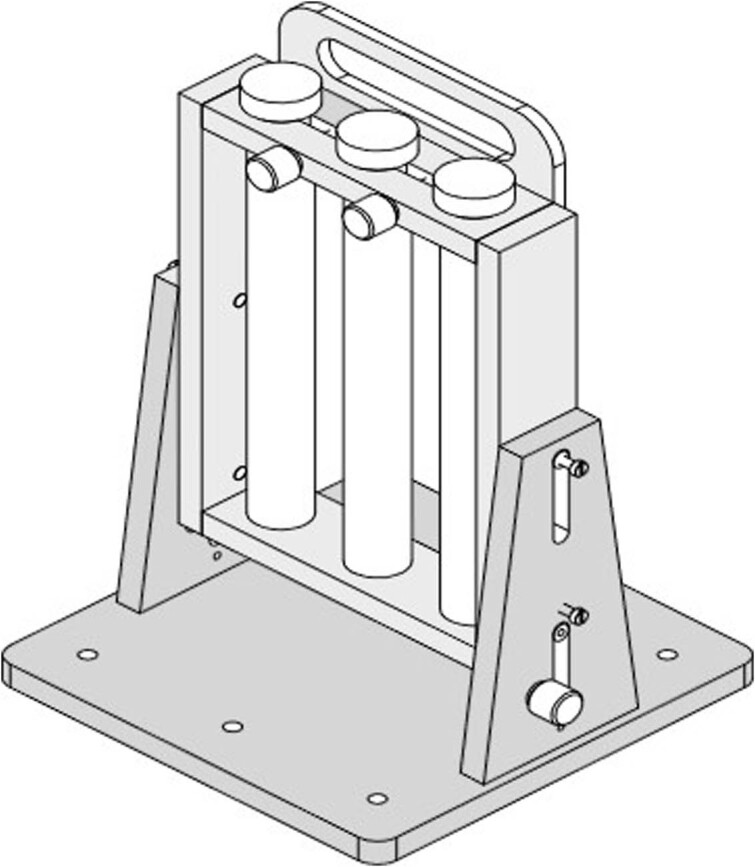
Schematic of the negative geotaxis gravity apparatus. Three cylindrical poly(methyl methacrylate) tubes were mounted on a support that could be lifted manually by the means of a handle.

**Extended Data Fig. 5:**
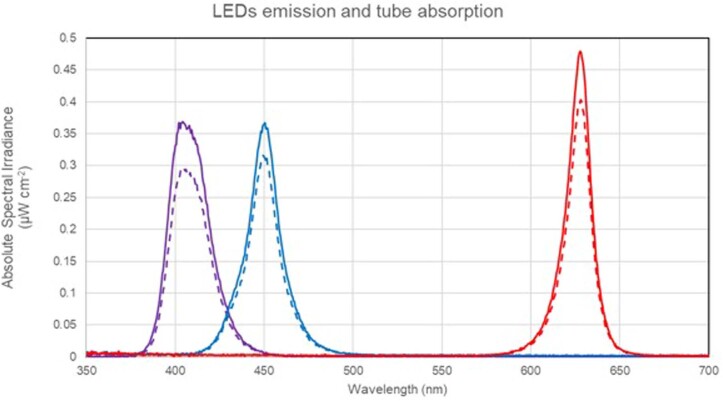
LED emission spectra. Emission spectra of the three LEDs arrays measured in front (continuous line) and behind (dashed line) the plastic tubes.

**Extended Data Fig. 6:**
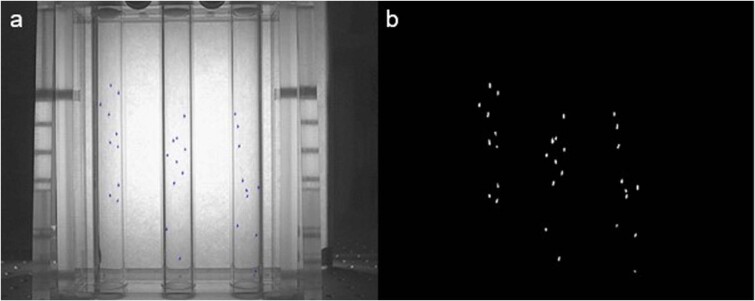
Video tracking of flies in the negative geotaxis gravity apparatus. **a**. Video frame of one of the experiments in the gravity apparatus. **b**. Visual representation of how the tracking program detected the flies in the tubes.

**Extended Data Fig. 7:**
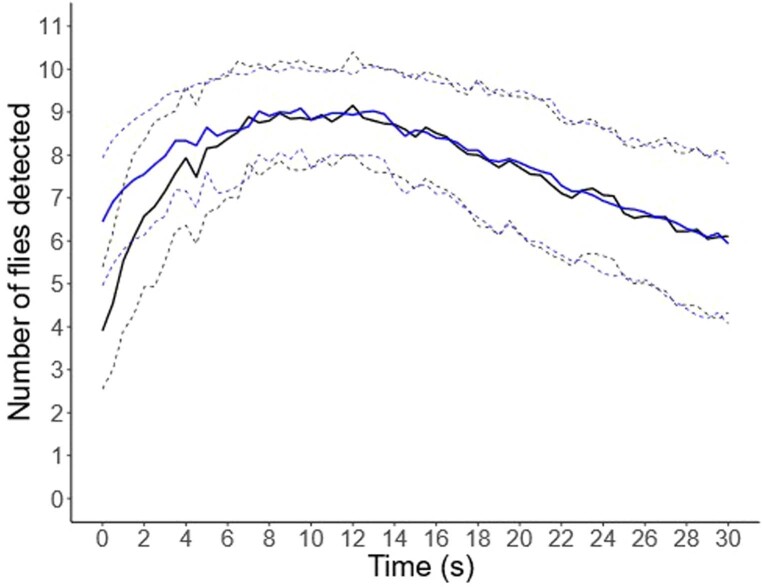
Comparison of manual and automated tracking in the negative geotaxis gravity apparatus. Three random videos were analysed by eye and then by the tracking software. The solid line shows the number of flies detected averaged over three randomly chosen videos with three tubes per video and ten flies per tube. Blue: counted by eye. Black: automatic counting. The dashed lines represent one standard deviation either side of the mean.

Following the negative outcome of the direct replication attempt, we decided to build an improved version of the original experiment and to conduct the tests in a more magnetically controlled environment^29^ than the original experiments. The new ‘gravity’ apparatus consisted of three clear plastic tubes, each containing ten flies, clamped to a stand which, when lifted vertically and released, caused the flies to be knocked down to the base of the tubes (Extended Data Figs. 4 and 5). This apparatus was placed in the middle of a double-wrapped, three-dimensional Merritt four-coil system (2 × 2 × 2 m^3^)^33^ placed inside one of our electromagnetically screened chambers^29^ (Fig. 3a). Movements of the flies inside the tubes were filmed and tracked automatically (Extended Data Figs. 6 and 7).

We used the same Canton-S line as in the original study (CS-LE) and performed the tests under dim blue light (410−490 nm) using a magnetic field of 300 µT. Additional experiments were conducted using magnetic fields of 0, 90 and 220 µT, each with its own sham exposure (Supplementary Fig. 7 and Supplementary Table 9). These additional experiments were motivated by a report of magnetic effects on geotaxis in flies tested in weaker fields than those used by Fedele et al.^9^ To evaluate climbing behaviour we first adopted the original criterion^9^ and determined the percentage of flies that climbed 15 cm in 15 s. We found that the percentage of ‘climbers’ did not differ significantly between field- and sham-exposed groups (Fig. 3b) at any of the magnetic field strengths studied (Supplementary Fig. 7). We further noted that such binary categorizations (climbers versus non-climbers), based on somewhat arbitrary cutoff criteria (minimum height, maximum time), were not robust but resulted in large scatter within both groups, simply because the observed distances climbed do not follow a bimodal distribution—that is, climbers and non-climbers (Supplementary Fig. 5). Therefore, to ensure that we had not missed a magnetic field effect, we analysed the average positions of the flies over time (Fig. 3c and Supplementary Fig. 6) but again found no evidence of a magnetic field effect on negative geotaxis.

Other studies have reported that flies are also magnetically sensitive when exposed to shorter wavelengths of light^6,18^. We therefore tested flies using ultraviolet (UV)-blue light-emitting diodes (LEDs; 380−450 nm) but found no effects of 0, 90, 220 or 300 µT magnetic fields (Supplementary Fig. 8, Supplementary Table 9 and Extended Data Fig. 5). In an attempt to recreate the environment of the laboratories in which the original experiments had been performed, we reintroduced the background radiofrequency fields that were absent in our electromagnetically shielded chamber. Yet again, no magnetic field effect was detected (Supplementary Fig. 9 and Supplementary Table 9). All of the above tests were repeated using a different Canton-S line (CS-OX; Supplementary Figs. 10–12 and Supplementary Table 10). In conclusion, we were unable to find any statistically significant magnetic field effect on *Drosophila* negative geotaxis (Supplementary Tables 6–8).

**Fig. 4:**
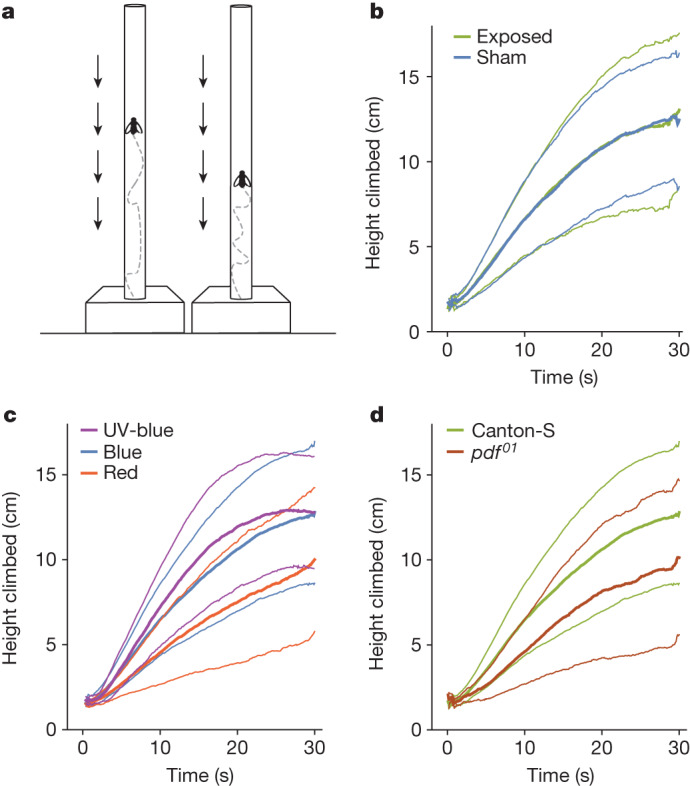
FlyVac negative-geotaxis assay. **a**, Schematic of the apparatus in which CS-LE flies were tested individually. Flies were exposed to a vacuum pulse that drew them down to the bottom of the tubes, after which they started to ascend. Arrows represent the effect of the vacuum pulse. **b**, Movements of individual flies were tracked and plotted as mean distance climbed as a function of time. Thick lines represent the mean behaviour of 53 flies under sham conditions and 49 flies in a 300 µT magnetic field, all in the presence of blue light (410–490 nm). There was no significant difference between exposed and sham conditions: analysis of variance (ANOVA) of linear mixed effect (LME), *P* = 0.9968. **c**, Positive control for light exposure; 308 flies were tested under UV-blue light (380–450 nm), 208 flies under blue light and 199 flies under red light (580−660 nm). On average, flies exposed to UV-blue and blue light climbed faster than those under red light: ANOVA of LME, *P* < 0.0001; UV-blue versus red, *P* < 0.0001; blue versus red, *P* < 0.0001. **d**, Positive control for negative geotaxis; 208 Canton-S and 82 *pdf*^*01*^ flies were tested under blue light. On average, Canton-S flies climbed faster than *pdf*^*01*^ flies: ANOVA of LME, *P* < 0.0001. **b**–**d**, Data shown as mean (thick lines) and mean ± s.d. (thin lines).

**Extended Data Fig. 8:**
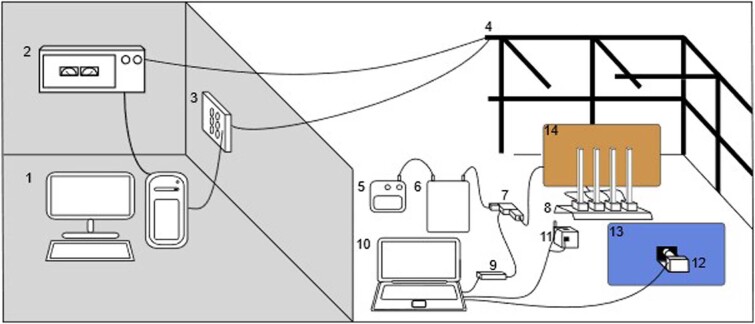
Schematic of the FlyVac apparatus. The currents in the coils were controlled by an external computer (1) via two data acquisition (DAQ) cards. The first (National Instruments, USB- 9263) connected the computer to the power supplies (2) (BOP 50-4M, Kepco Inc.) and controlled the magnitudes of the currents in the coils. The second (National Instruments USB-6051) controlled the switching box (3) responsible for the directions of the currents (parallel or antiparallel) through the windings of each coil (4). In the schematic, most of the 12 coils were omitted for clarity. A picture of the chamber and the coils is available in Schwarze *et al*. ^29^ A MATLAB script controlled the two DAQ cards (a) to provide an analogue output to the power supply and (b) to switch the direction of the currents via a digital output. Other components (described above): vacuum pump (5), buffer tank (6), solenoid valve (7), FlyVac apparatus (8), relay (9), and a laptop computer (10) that controlled the valve, sensors (11) and video camera (12). One LED plate (13) illuminated the tubes; the other (14) provided an infrared background for the video camera. Components 7–14 were placed on a table in the middle of the coils; components 5 and 6 were as placed as far as possible away from the coils.

**Extended Data Fig. 9:**
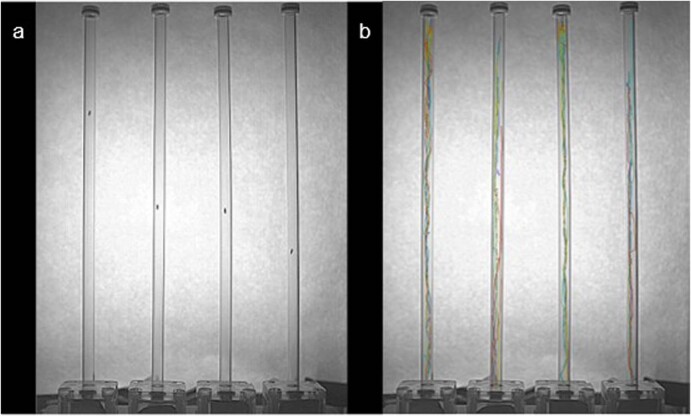
Video tracking of flies in the negative geotaxis FlyVac apparatus. **a**. Video frame of one of the experiments in the FlyVac set-up. **b**. Visual representation of how the tracking program detected the flies in the tubes. The different colours showed the different tracks performed by a single fly in the five repetitions.

Finally, to obtain robust climbing data that were not potentially confounded by group effects, we redesigned the geotaxis assay to allow monitoring of single flies rather than groups of ten so that individual trajectories could be recorded and analysed. Inspired by the FlyVac apparatus of Kain et al.^34^, we used a brief reduction in air pressure to draw each fly down to the bottom of its tube and then filmed its subsequent ascent (Fig. 4a and Extended Data Fig. 8). The measurements were fully automated and, once again, conducted in a blinded fashion to avoid any possible bias. Flies were tested in 0, 90, 220 and 300 µT magnetic fields, with an equal number of sham controls, using the same Merritt coils in the same shielded chamber as before, tracking the movement of each individual fly in five separate trials (Extended Data Fig. 9).

**Extended Data Fig. 10:**
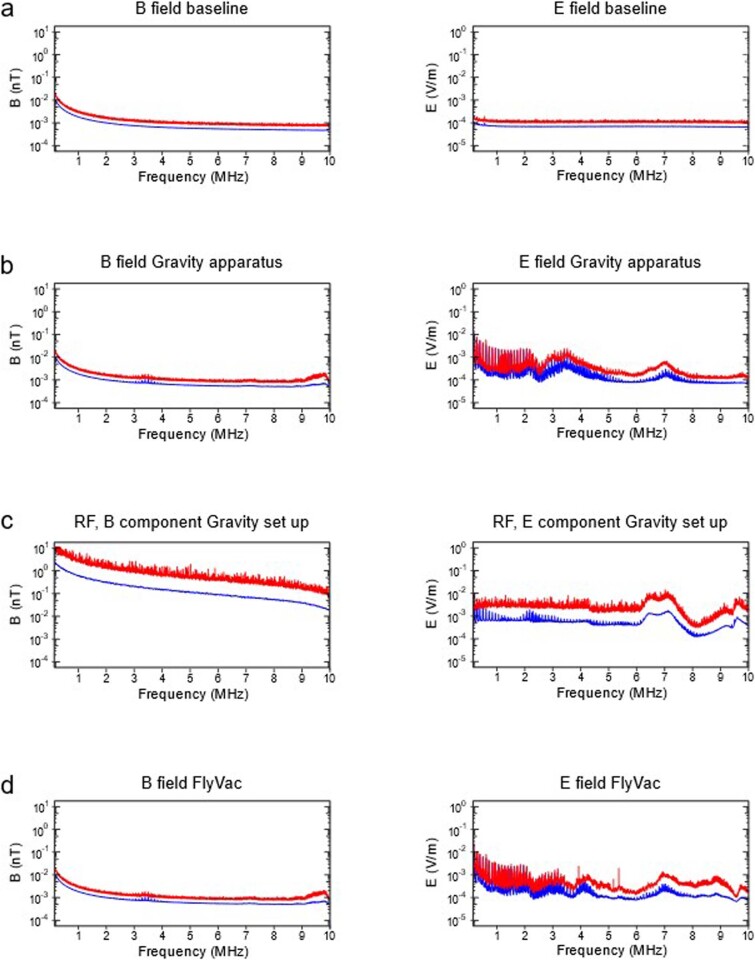
Electromagnetic field spectra. Blue line: average field intensity; red line max-hold intensity. **a**. Magnetic (left) and electric (right) components of the time-dependent fields measured when the behavioural set-ups were switched off. These measurements were considered as baselines. **b**. Magnetic (left) and electric (right) components of the time-dependent fields measured when all the gravity apparatus electrical devices (LED plates, laptop, camera and sensors) were switched on. **c**. Magnetic (left) and electric (right) components of the time-dependent fields measured when all the gravity apparatus electrical devices (LED plates, laptop, camera and sensors) were switched on and broadband RF was reintroduced into the experimental chamber. **d**. Magnetic (left) and electric (right) components of the time-dependent fields measured when all the FlyVac electrical devices (LED plates, laptop, camera, sensors, vacuum pump, valve and relay) were switched on.

We tested single flies under UV-blue (380−450 nm), blue (410−490 nm) and red (580−660 nm) light and compared wild-type flies with a mutant, *pdf*^*01*^, known to have a negative-geotaxis deficiency^35,36^. As expected, Canton-S flies (CS-LE) climbed faster under UV-blue and blue light than under red light and faster than *pdf*^*01*^ flies under blue light (Fig. 4c,d). Although these positive-control experiments produced results similar to those of the original study, after testing 1,960 flies individually we found no magnetic field effect regardless of *Drosophila* strain, wavelength or magnetic field intensity (Fig. 4b, Supplementary Figs. 13–15 and Supplementary Table 11). In contrast to the original experiments of Fedele et al.^9^, our experiments were performed in a completely non-magnetic research facility^29^ in which both static and time-dependent magnetic fields could be meticulously controlled (Extended Data Fig. 10), with much larger sample sizes and with all experiments carefully blinded.

Even though millitesla magnetic field effects on purified *Drosophila* cryptochrome have been observed in vitro^37^, the magnetic sensitivity of a protein is not sufficient evidence for magnetic sensing in the whole organism. To demonstrate this, a widely reproducible test showing magnetically guided behaviour would be required—especially because *Drosophila* has no obvious need for a magnetic compass, being neither a true migrant nor a central-place forager^38^. Although *Drosophila* does engage in long-range, wind-assisted dispersal, there is no convincing evidence that it exhibits regular, seasonally reversed, long-range migrations in specific compass directions^39,40^.

Having tested a total of 108,609 *Drosophila* from different strains over a period of 6 years under extremely carefully controlled conditions, having seen clear results in the positive-control experiments and having employed robust statistical analyses, we conclude that (1) *Drosophila* has no naive preference for or against a magnetic field and cannot be trained to associate a sucrose reward with a magnetic field using the protocols of Gegear et al.^6,7^ and Foley et al.^8^; and (2) that *Drosophila* negative geotaxis is not affected by external magnetic fields when using the protocol of Fedele et al.^9^ We are aware that there are other reports of magnetic field effects on *Drosophila* behaviour that we have not attempted to replicate (for example, refs. ^10–23^). However, (1) these studies share characteristics of Gegear et al.^6,7^, Foley et al.^8^ and Fedele et al.^9^; (2) they involved much smaller sample sizes than those used here; and (3) the static and time-dependent magnetic environments were not as meticulously controlled as in our experiments. Considering our large-scale replication effort, we seriously doubt whether *Drosophila* can sense near-Earth-strength (below 500 μT) magnetic fields at all and thus strongly suggest that night-migratory songbirds remain the organism of choice for elucidating the mechanism of light-dependent magnetoreception.

## Methods

### Non-magnetic laboratory

All experiments were conducted in one of the chambers of the electromagnetically shielded laboratory at the University of Oldenburg (described in detail in ref. ^29^). The building was constructed from non-magnetic materials and each of the three rooms was individually electromagnetically screened (S101, ETS Lindgren) with attenuation factors of 10^5^ at 10 kHz and better than 10^6^ at frequencies above 150 kHz. The geomagnetic field penetrates the aluminium shielding without distortion.

### *Drosophila* strains

*Drosophila* stocks were reared in culture vials with standard maize. The genotypes of the flies tested were Canton-S (referred to as CS-OX), *w*;Canton-S (referred to as CS-LE), *pdf*^*01*^, Canton-S M, *w*^*1118*^ and *w*^*1118*^GR. The CS-OX line was provided by S. Waddell (Centre for Neural Circuits and Behaviour, University of Oxford, UK); CS-LE and *pdf*^*01*^ by C. P. Kyriacou (Department of Genetics, University of Leicester, UK); and Canton-S M, *w*^*1118*^ and *w*^*1118*^GR by E. M. C. Skoulakis (Biomedical Sciences Research Centre Alexander Fleming, Vari, Greece).

Vials were kept at 25 °C in incubators in which a 12/12 h light/dark regime was maintained. The light was switched on at 08.00. Experiments were run at 25 °C.

### T-maze assay

#### Apparatus

The binary-choice T-maze was an exact replica of that used by Gegear et al.^6^, constructed entirely from non-magnetic materials according to a blueprint provided by S. M. Reppert (University of Massachusetts Medical School, USA). The apparatus consisted of two double-wrapped magnetic field coils (100 mm diameter) and a poly(methyl methacrylate) construction that included an elevator to transport the flies between the training and testing tubes. The tubes were made of polystyrene (100 mm in length, 14 mm inner diameter, 16 mm outer diameter) with rounded bottoms. This apparatus could be placed with either the training tube pointing towards one of the coils or with one testing tube within each coil so that the flies could be exposed to the same light and magnetic stimuli during the training and testing phases of the experiment. The T-maze apparatus and coils were placed inside a wooden box with the inner walls painted black to minimize visual cues. The box was lit by two striplights, a Zoo Med Reptisun 10.0 UVB, 18 W and a JBL FullspectrumNatur, 18 W. The lights were positioned directly above the T-maze apparatus, 44 cm from the tubes. Experiments were performed under either exposed (currents flowing in parallel through one coil and antiparallel in the other) or sham (no current in either coil) conditions. A photograph of the setup is shown in Extended Data Fig. 1.

#### Experimental procedure

The experimental procedure followed that of Gegear et al.^6^ It consisted of three 2 min phases—acclimatization, training and testing—with a 1 min rest in between. The acclimatization phase allowed the flies to become familiar with the apparatus. Approximately 100 flies were loaded into an empty tube attached to the apparatus, with the tube pointing towards one of the coils in which no current flowed. After 2 min acclimatization the flies were transferred to the elevator and kept there for 1 min.

The acclimatization tube was replaced by a training tube that contained the sucrose reinforcement. During the training phase the flies could feed for 2 min while exposed to a magnetic field with an intensity of about 500 µT at the end of the tube. Flies were then carefully transferred back into the elevator and held there for 1 min. The coils were then switched off so that flies were exposed to the Earth’s magnetic field only. During this time the training tube was removed and two empty tubes were attached to the elevator, thus forming the T-maze for the testing period.

In the testing phase the flies had a choice between two tubes, one of which provided a magnetic field of around 500 µT. The elevator slider was opened and the magnetic field switched on at the same time. After 2 min the T-maze was blocked by reversing the slider so that the flies in each tube could be counted. As reported in the original study^6^, the naive flies were tested using a different protocol (naive-1 in Fig. 1). One set of around 100 flies was loaded directly into the elevator section of the horizontally placed choice chamber and the magnetic field was turned on. Flies were transferred into the T-port after 1 min and kept there for a further 2 min. In addition we tested the flies again in the naive condition, only this time we followed exactly the same protocol used for the trained ones except that the training tube contained a piece of Whatman filter paper but no sucrose (naive-2 in Fig. 1).

Male and female flies, up to 5 days old, were used. Flies were starved for 22 h before the test and were provided with a 1% agar solution inside the vials. The reward consisted of a dried Whatman filter paper that had previously been soaked in a saturated sucrose solution.

All experiments were conducted with the experimenter blind to the magnetic conditions (exposed versus sham and magnetic field in the left versus right arm of the maze).

#### Odour-conditioning experiments

As a positive control we tested the flies’ ability to associate an odour (octan-3-ol (OCT) or 4-methylcyclohexanol (MCH)) with the sucrose reward using protocols similar to those in the magnetic conditioning experiments.

A total of 100–150 flies, up to 5 days old, were starved for 18–24 h in a vial containing a layer of 1% agar and a piece of dried filter paper. OCT and MCH were diluted 1:1,000 in mineral oil. The sucrose reward was a 2 g ml^−1^ sucrose solution dried overnight on a filter paper such that a uniform layer of crystallized sugar was formed.

In our two-odour discrimination training procedure, flies were given the opportunity to feed on the sucrose solution (unconditioned stimulus) in association with the odour that was to become the reinforced conditioned stimulus (CS^+^) while the other odour, paired with an empty piece of filter paper, was to become a non-reinforced stimulus (CS^−^). In detail, flies were first exposed for 2 min to the CS^−^ odour presented with a dry filter paper, followed by 30 s rest, then they were transferred to another tube with a filter paper impregnated with dried sugar and presented with the CS^+^ odour for 2 min. Following this training they were transported in an elevator to the choice point in the T-maze, where they were given 2 min to choose between the two odours presented during training. A different odour (OCT versus MCH) was pumped into each arm. If the flies had learned to associate the CS^+^ odour with the sucrose reward, they would choose the arm with that odour presented. For each experiment, two T-mazes were run simultaneously. In one the flies had been trained to associate OCT with the sucrose reward and, in the other, MCH (Extended Data Fig. 2). After each experiment the CS^+^ odour in each T-maze is switched (that is—if, in the left machine the CS^+^ was OCT, it was replaced by MCH in the repeat, and vice versa).

These experiments were performed by M. Bassetto in the laboratory of S. Waddell (Centre for Neural Circuits and Behaviour, University of Oxford). We are very grateful to him and to members of his research group for advice and guidance.

#### T-maze data

All statistical analyses were performed in R (https://www.r-project.org/). We evaluated the T-maze binary-choice data following the procedure reported by Gegear et al.^6^ For each set of flies (around 100 each) a preference index (PI = 2*P*_M_ – 1) was calculated, where *P*_M_ is the proportion of flies in the arm of the maze with the magnetic field. Initially we analysed data using the approach reported in Gegear et al.^6^ (*t-*test and ANOVA). We then reanalysed the data by applying a general linear model with binomial error structure appropriate for proportional data^41^. Cohen’s effect size, *h*, was calculated to estimate the sample size required to achieve the significance threshold of *P* < 0.05 for a given effect size. For details on the analyses used, see Supplementary Information 1.1.

### Negative-geotaxis and gravity assay

#### Original apparatus

The original apparatus used by Fedele et al.^9^ was made available by C. P. Kyriacou. It consisted of an aluminium box containing two double-wrapped coils with 50 windings, each capable of producing a magnetic field of roughly 300 or 500 µT. The flies were knocked down to the bottom of plastic vials by means of a ‘swinger apparatus’, which ensured that the vials were moved simultaneously and with equal force. The experiments were filmed with an infrared camera (Logitech). Flies were tested under either dim blue or red light produced by strips of LEDs. The lights had an intensity of 0.25 μW cm^−2^ measured on the tube surface (Extended Data Fig. 3).

#### Testing procedure

Ten 2–3-day-old flies were loaded into plastic vials, which were placed in the swinger apparatus. Three vials were tested simultaneously. The flies were knocked to the bottom of the vials and those that were able to reach a height of 15 cm in 15 s were considered to be climbers. Each tube was tested ten times, with 30 s between repeats. After the first five trials, flies were allowed to rest for 15 min after which they were tested five more times. The order in which sham and exposed conditions were tested was randomized. For the sham condition, antiparallel currents flowed in both double-wrapped coils.

#### Gravity apparatus

Negative geotaxis was also studied using an experimental arrangement designed to resemble that of Fedele et al.^9^ As shown in Extended Data Fig. 4, three cylindrical poly(methyl methacrylate) tubes (length 200 mm, inner diameter 20 mm, outer diameter 25 mm) were mounted on a support constructed of non-magnetic materials. The support could be lifted manually through 6 cm, by means of a handle, and then released to knock the flies down to the base of the tubes. A thin layer of rubber beneath the tubes acted as a shock absorber to reduce recoil. The apparatus was made entirely of Delrin and poly(methyl methacrylate) to avoid any distortion of the applied magnetic field. It was built in the mechanical workshop of the Department of Chemistry of the University of Oxford.

The wavelength-dependence of the flies’ ability to climb was investigated by uniformly illuminating the tubes using one of three purpose-built arrays of LEDs: UV-blue (380−450 nm, LHUV-0405-0600 Ultraviolet LUXEON Z LED), blue (410−490 nm, LXZ1-PR01 Royal-Blue LUXEON Z LED) or red (580−660 nm, LXZ1-PD02 Red LUXEON Z LED). Each array plate had 20 LEDs separated from each other by 45 mm. The arrays were built in the electronics workshop of the Department of Chemistry of the University of Oxford. The LED plates were supplied with d.c. current by a power supply unit (Manson SPS 9400). The spectral distributions of the three diode types in the range 300−700 nm were measured using a Maya2000 Pro spectrometer (Ocean Optics) with an integration time of 100 ms. Measurements were made both in front of and behind the tubes to check for differential absorption at different wavelengths. Apart from some minor wavelength-independent attenuation, probably due to light scattering, no difference was detected. The light incident on the tubes was approximately 0.25 µW cm^−2^ and therefore comparable to that used by Fedele et al.^9^ (Extended Data Fig. 5).

The flies were filmed using an infrared video camera (Thorlabs DCC1645C) at ten frames s^−1^. An infrared filter (Schott RG 780 filter, 50 × 50 mm^2^) was positioned in front of the camera and the tubes were backlit with a purpose-built plate of infrared LEDs (850 nm or above; L1IZ-0850000000000 Infrared LUXEON IR LED). The LED plate had 20 LEDs separated from each other by 45 mm. In the videos the flies appeared black against a white background.

As a further control we installed a magnetic sensor (SparkFun Triple Axis Magnetometer Breakout HMC588L), a light sensor (Adafruit TSL2591 High Dynamic Range Digital Light Sensor) and a temperature sensor (Maxim Integrated DS18B20). These were controlled by an Arduino-based data acquisition system with the Arduino board enclosed in an electrically shielded box. The sensors were placed on a poly(methyl methacrylate) cube situated close to the tubes, just out of view of the camera. During behavioural experiments the three sensors sampled at 1 Hz to check the constancy of environmental conditions, and to provide feedback on the proper functioning of the magnetic field-exposure equipment, without showing the exposure conditions to the experimenter. The gravity setup, LED panels and sensors were also placed inside the double-wrapped, three-dimensional Merritt four-coil system.

A LabVIEW programme allowed control of the camera and sensors. The information recorded by the magnetic field sensor was encrypted and shown only after completion of data analysis. In this way the experimenter was blind to magnetic field condition. A transmission control protocol/Internet protocol server programmed in MATLAB was used to synchronize data acquisition on the laptop (inside the shielded room) with the desktop computer (outside the room) that controlled the Merritt coils. Synchronization was independently verified using the magnetic field registered by the HMC588L sensor. Thus, although hidden from the experimenter, the exposure settings were always traceable. A schematic of the setup is shown in Fig. 3a.

#### Experimental procedure

Ten flies were gently loaded into each of the three tubes and allowed to rest for at least 5 min before the tubes were clamped to the support. After a 5 min rest the flies were pre-exposed to the light and magnetic stimulus for 120 s. In each experiment the support was lifted and released five times in quick succession to knock the flies to the base of the tubes, after which they were filmed. This was repeated four times at 30 s intervals to give a total of five trials. After each set of five measurements the tubes were washed with 70% ethanol solution. Each group of flies was tested under a single magnetic condition, either sham or exposed depending on the direction of the currents through the windings of the Merritt coils.

Only male flies (1−3 days old) were tested. These were collected at least 24 h before the experiments after being anaesthetized on ice and kept in incubators until testing time. Tests were performed between 13.00 and 17.00. Four magnetic field intensities were used: 0, 90, 220 and 300 µT. For each magnetic field a sham test was performed (with antiparallel currents through the coil windings). A single set of experiments therefore comprised eight separate magnetic conditions. Each set of experiments was repeated five times, giving a total sample size of 15 independent biological replicas per condition. In each set of experiments the order of sham and exposed conditions was randomized and blinded so that it was impossible for the experimenter to know the magnetic field conditions experienced by the flies.

#### Negative-geotaxis video analysis

Every experiment was recorded at 1,280 × 1,024 pixel resolution and the videos were analysed and tracked. All videos were first edited using Fiji (http://fiji.sc/Fiji; ref. ^42^) to produce high-contrast images on which it was easier to spot the flies inside the tubes. We corrected for the gamma (value, 0.45) and sharpened the image (unsharp mask-radius sigma, 1.0; mask weight, 0.93).

The flies in the tubes were tracked using a MATLAB script to record their positions in each frame. To this end, a representative background image (that is, a still image of the exposure apparatus without flies) was calculated from the entire recording. This was realized in a two-pass process. In the first step, the arithmetic average of all recorded frames was calculated. In a second iteration, a refined background value was calculated for each pixel individually by considering only those frames for which pixel intensity did not fall short of the average from the first iteration by more than 20% (presumably due to the presence of a fly). Pixels with contributions from fewer frames than corresponded to the total length of the recording were not considered reliable. Instead, these pixels were filled in from neighbouring pixels based on the moving average with a window size of ten pixels. The image so obtained was convoluted with a two-dimensional Gaussian with standard deviations of one pixel to obtain a faithful and smoothed representation of the background—the scene without flies. For the tracking, each frame was linearly scaled in intensity to match the background image in several regions inaccessible to the flies—in between the tubes. The scaled image was then subtracted from the background to obtain a raw representation of moving flies as bright pixels against a dark background. This raw image was subject to morphological opening using a disk of size comparable to the flies as structuring element. The dimensions of the disk were delimited as follows: major axis range between four and 20 pixels, minor axis between three and 20 pixels, surface of the disk between eight and 200 pixels. Binarization with a suitable threshold, followed by the removal of small connected regions, yielded a representation of flies as white regions against a black background. The centroid, area and semimajor and semiminor axes of these regions were calculated. Provided that the geometrical measures fitted the expected range, the centroid was recorded for further analyses. Regions that did not meet these criteria were disregarded (Extended Data Fig. 6).

To check the reliability of the tracking programme in the gravity apparatus assay, three random videos were chosen and the number of flies in each tube was counted visually every five frames (that is, every 0.5 s). The lack of proper resolution in the first 4 s of each video is mainly due to the difficulty in tracking single flies when close to each other, and also due to reflections of the flies on the plastic surface of the tube (Extended Data Fig. 7). All these issues were solved once flies were tested and tracked individually in the FlyVac apparatus (see Methods section ‘FlyVac assay’).

In the gravity apparatus all frames that detected more than ten flies were excluded from the statistical analysis and considered as false positives. In the FlyVac setup, the data from any fly that was not sucked to the bottom by all five vacuum pulses, or that showed no mobility in all five trials, were discarded.

#### Gravity apparatus data

In each experiment three tubes containing ten flies each were tested in five consecutive trials, 30 s per trial. Before each trial, flies were manually tapped on the table five times to knock them to the bottom. This experiment was repeated with new flies for every magnetic field treatment (four magnetic field condition plus four corresponding shams equals eight treatments). Each set consisted of eight treatments and was replicated five times.

All statistical analyses were performed in R. To ensure correct replication of the original study we adopted the statistical test used in Fedele et al.^9^ We initially evaluated climbing behaviour as the percentage of flies that climbed 15 cm in 15 s and compared this ratio of climbers between experimental groups with repeated-measurements ANOVA. To ensure that we did not overlook a possible effect, we additionally analysed the difference in the climbing ratio of flies over the whole period of the trial rather than just at 15 s. We applied a generalized linear mixed model to account for the binomial error structure of our data (climbing ratios) and for the repeated measurements (consecutive video frames). The random part of the model was (1|id) + (1|trial/frame), where ‘id’ is unique to each fly. ANOVA (from the package ‘car’) was then used to estimate the *F* and *P* values for the factor ‘Exposure’:

$$\begin{array}{l}{\rm{prop.glme}}={\rm{lme}}4::{\rm{glmer}}({\rm{cbind}}({\rm{n}},{\rm{sum.n}}-{\rm{n}})\\ \, \sim {\rm{Exposure}}+(1| {\rm{id}})+(1| {\rm{trial}}/{\rm{frame}}),{\rm{data}}={\rm{df}},\\ \,{\rm{family}}={\rm{binomial}},{\rm{na.action}}={\rm{na.exclude}})\end{array}$$

$${\rm{Anova}}({\rm{prop.glme}},{\rm{type}}= \mbox{``} {\rm{III}}\mbox{''}).$$

To further analyse negative-geotaxis behaviour we estimated the actual position of flies during trials. Because it was impossible to track every single fly independently of the others, we averaged the position of ten flies per video frame (0.1 s) and then applied a LME model to analyse the effects of both magnetic field condition (0, 90, 220 or 300 µT) and exposure (sham versus exposed), as well as those of the interaction of these two factors on the average distribution of flies over time. The random part of the model was (1|ID) + (1|trial/frame), where ‘ID’is unique for each independent biological replicate (tube with flies):

$$\begin{array}{l}{\rm{fly.lme}}={\rm{lmerTest::lmer}}({\rm{Ycm}} \sim {\rm{Exposure}}\\ \,\times \,{\rm{condition}}+(1|{\rm{ID}})+(1|{\rm{trial}}/{\rm{frame}}),{\rm{data}}={\rm{df}},\\ \,{\rm{na.action}}={\rm{na.exclude}})\end{array}$$

The LME model of the same structure was also applied to every independent set of experiments, consisting of eight experimental treatments (four magnetic field conditions × two exposures).

ANOVA was then used to estimate *F* and *P* values for each of the three factors (condition, exposure and exposure:condition) in every LME model.

### FlyVac assay

#### Apparatus

The FlyVac apparatus (inspired by Kain et al.^34^) constructed to study *Drosophila* negative geotaxis comprised four vertical cylindrical polystyrene tubes (length 200 mm, inner diameter 5 mm, outer diameter 6 mm). Each tube was closed at the top by means of a small cap with air holes and connected at its base to a vacuum pump via a solenoid valve. Each tube contained one fly. When the valve was opened, the air vortex quickly and safely whisked the flies to the bottom of the tubes. On closing the valve after 3 s, the flies started to climb inside the tubes. The connections between the bottom of each tube and the vacuum system were stereolithographically printed using epoxy resin, to avoid any distortion of the local magnetic field. The solenoid valve (SMC VT 307v-50Z1-01F-Q) was connected to the vacuum pump (Vacuubrand, ME4CNT) via a tank (with an approximate volume of 30 l to ensure that the flies were subject to a reproducible reduction in pressure.

The experiment used the same LED panels, sensors, camera, laptop and LabVIEW programme as in the gravity experiments. The experiment was completely automated to remove any possible artefact due to human interaction. As in the previous experiment, the camera and sensors were connected to a laptop via USB cables. The solenoid valve was connected and controlled by a relay (Four Channel USB Relay Module, Numato Lab), which was connected to the laptop via a USB cable. The laptop controlled the camera, sensors and relay. The laptop and relay were placed in a grounded aluminium box inside the shielded room, as far as possible from the coils (Extended Data Fig. 8).

Preliminary FlyVac experiments were performed at the Centre for Neural Circuits and Behaviour at the University of Oxford under the guidance of S. Waddell, whom we thank for extensive advice and laboratory facilities, in addition to technicians in the CNCB workshop who designed and constructed the apparatus.

#### Experimental procedure

A single fly was transferred into each of the four FlyVac traps (formed by the base and plastic tube), where they rested for 120 s before the FlyVac traps were connected to the apparatus inside the screened chamber. Flies were then pre-exposed for 120 s to the magnetic field and light conditions under which they would be tested. At 30 s intervals flies were sucked down to the base with a 3 s vacuum pulse; this was repeated another four times to give a total of five trials. Each fly was tested under a single magnetic condition. All experiments were filmed and tracked as described in Methods Section ‘Negative-geotaxis video analysis’ (Extended Data Fig. 9). After each experiment, the tubes were washed with a 70% ethanol solution.

The gender of the flies, keeping and collecting, daily time of the experiments, magnetic field conditions, blinding and randomization of the experiments were the same as described in the ‘Negative-geotaxis and gravity essay’ section.

#### FlyVac data

In each experiment, four tubes containing one fly each were tested in five consecutive trials, 30 s per trial. Before each trial flies were sucked to the bottom of the tube by the means of a 3 s vacuum pulse. This experiment was repeated with new flies for every magnetic field treatment (four magnetic field condition plus four corresponding shams, totalling eight treatments). For every magnetic field treatment roughly 50 flies were tested. The climbing behaviour of individual flies in the FlyVac setup was analysed as the absolute position of those flies over time. We applied a LME of the same structure as for the gravity setup data, followed by ANOVA to test for the effect of different magnetic field treatments. For the positive control of the climbing behaviour itself we included the factor ‘genotype’/‘light condition’ into the fixed part of the model.

### Static magnetic fields

#### Description of coils

The static magnetic fields in all negative-geotaxis experiments were generated by a double-wrapped, three-dimensional Merritt four-coil system of dimensions 2 × 2 × 2 m^3^ (ref. ^29^). Experiments were performed in the centre of the coils, where field homogeneity was better than 99% (ref. ^43^). Currents in the coils ran through subsets of windings in either parallel or antiparallel direction. When the currents were antiparallel the flies experienced the normal geomagnetic field of Oldenburg (53.152437° N, 8.164159° E) (sham exposure, 48.3 µT intensity, 67.7° inclination). When the currents ran parallel the flies were exposed to four magnetic fields of varying total intensity: 0, 90, 220 and 300 µT (which was the highest producible field without risking damage to the coils). Apart from the 220 µT experiments, in which the magnetic field was applied in the horizontal plane (to mimic the conditions of the original report^9^), the magnetic field was applied on the vertical (*z*) axis.

#### Measurement of static magnetic fields

In the T-maze assay the applied magnetic field was measured using an F. W. Bell Gaussmeter (Model 5170) with a 4-in standard probe (STH17-0404). For antiparallel currents, no measurable deviation from the geomagnetic field was detectable.

In negative-geotaxis assays, magnetic fields were measured with either a FVM 400 Vector Magnetometer (Meda; for fields below 100 µT) or a Model 475 DSP Gaussmeter (Lake Shore Cryotronics; for stronger fields). For fields below 100 µT it was possible to measure the *x*, *y* and *z* components whereas for higher fields only total intensity could be measured.

### Time-dependent electromagnetic fields

Spectra of the electromagnetic fields in the immediate neighbourhood of the behavioural apparatus were measured when all electronic components were switched off (as a control) and during an experimental trial (when all equipment was running), using a signal analyser (Rohde and Schwarz, FSV 3 Signal and Spectrum Analyzer, 10 Hz–3.6 GHz). The magnetic components were measured using a calibrated active-loop antenna (Schwarzbeck Mess-Electronik, HFS 1546) between 150 kHz and 10 MHz. The electric components were measured using a calibrated active biconical antenna (Schwarzbeck Mess-Electronik, EFS 9218), between 9 kHz and 10 MHz, as described in Engels et al.^26^ (Extended Data Fig. 10a,b,d).

The electromagnetic shielding of the room in which all experiments were performed very effectively excluded anthropogenic time-dependent fields, with attenuation factors of 10^5^ at 10 kHz and above 10^6^ at frequencies above 150 kHz. To check whether the absence of this background electromagnetic noise affected the flies’ ability to respond to static magnetic fields, in one experiment we introduced broadband electromagnetic noise in a range from about 2 kHz to about 10 MHz (20 V peak-to-peak, 7 Vrms, 13 nT total field rms) by means of a passive-loop antenna (ETS Lindgren EMCO antennas, Model 6511, 20 Hz–5 MHz) placed close to the gravity apparatus (Extended Data Fig. 10c). As described in Schwarze et al.^29^, the antenna was driven by a RIGOL, DG1022 signal generator.

### Reporting summary

Further information on research design is available in the Nature Portfolio Reporting Summary linked to this article.

## Online content

Any methods, additional references, Nature Portfolio reporting summaries, source data, extended data, supplementary information, acknowledgements, peer review information; details of author contributions and competing interests; and statements of data and code availability are available at 10.1038/s41586-023-06397-7.

### Supplementary information

Supplementary Information The Supplementary Information file has two main sections (T-maze assay and negative-geotaxis assay) with additional references, and contains 15 figures and 11 tables showing results and statistics of magnetic-sensing measurements.

Reporting Summary

## Data Availability

All source data are available at 10.17605/OSF.IO/HZ98Q.

## Code availability

All codes used for data analysis (in R) are available at 10.17605/OSF.IO/HZ98Q.
