# Supplementary material for: No evidence for magnetic field effects on the behaviour of Drosophila
Source: Nature. 2023 Aug 9;620(7974):595–9. doi: 10.1038/s41586-023-06397-7 (PMC10432270; doi:10.1038/s41586-023-06397-7)
Supplement: Supplementary file 1 — The Supplementary Information file has two main sections (T-maze assay and negative-geotaxis assay) with additional references, and contains 15 figures and 11 tables showing results and statistics of magnetic-sensing measurements. [file 41586_2023_6397_MOESM1_ESM.pdf]

nature portfolio

https://doi.org/10.1038/s41586-023-06397-7

Supplementary information

No evidence for magnetic field effects on the behaviour of *Drosophila*

In the format provided by the authors and unedited

No evidence for magnetic field effects on the behaviour of *Drosophila*

Marco Bassetto, Thomas Reichl, Dmitry Kobylkov, Daniel R. Kattnig, Michael Winklhofer, P. J. Hore, Henrik Mouritsen

Corresponding authors: henrik.mouritsen@uni-oldenburg.de
peter.hore@chem.ox.ac.uk

Supplementary Information

Table of Contents

1. T-maze assay 2
1.1 Statistical considerations on Gegear *et al.*[6] 2
1.2 Supplementary results 6
1.2.1 Statistical analysis 6
1.2.2 Tests with lower sucrose concentrations 6
1.2.3 Tests under different conditions 8
1.2.4 Summary of T-maze experiments 10
1.3 Odour conditioning experiments 10
2. Negative geotaxis assay 11
2.1 Statistical considerations on Fedele *et al.*[9] 11
2.2 Figure 2 climbing statistics 14
2.3 Gravity apparatus supplementary results 17
2.3.1 Canton-S LE Blue light 17
2.3.2 Canton-S LE UV-blue light 18
2.3.3 Canton-S LE Blue light and radiofrequency exposure 19
2.3.4 Summary 20
2.3.5 CS- OX Blue light 22
2.3.6 CS- OX UV-Blue light 23
2.3.7 CS- OX UV-Blue light and radiofrequency exposure 24
2.3.8 Summary 25
2.4 FlyVac apparatus supplementary results 27
2.4.1 Canton-S LE UV-blue & blue light 27
2.4.2 Canton-S LE Red light & pdf^{01} blue light 28
2.4.3 Canton-S OX Blue light 29

2.4.4 Summary 30
Supplementary References 31

1. T-maze assay

1.1 Statistical considerations on Gegear *et al.*[6]

In the original study by Gegear *et al.*[6] the data from the T-maze experiments were reported as a Preference Index, $PI = 2P_M - 1$, where P_M is the proportion of flies in the arm of the maze with the magnetic-field (Fig. S1A). This linear transformation from P_M to PI is, however, redundant, since it does not change the binomial nature of the original data, which were based on a binary counting scheme. As a result, both PI and P_M are proportional variables strictly bound by the intervals [–1, +1] and [0, 1], respectively. The distribution of errors, and hence the error margins, of proportion data differs markedly from the normal error distribution of continuous data[1]. The error margins, in turn, define the confidence limits for the estimated mean proportion.

This seemingly minor issue turns out to be critical for the statistical analysis. By performing parametric tests (Student *t*-test and ANOVA), Gegear *et al.*[6] neglected the binomial nature of their data and treated them as if they were normally distributed and continuous. As a result, the authors reported a highly significant effect of the magnetic field on the flies' behaviour (see, for example, Fig. 1b, wild-type Canton-S, in Gegear *et al.*[6]), which most likely is a "false positive" result.

We verified this by re-analyzing the largest magnetic field effect reported in Gegear *et al.*[6], considering the error margins for proportion data. To do so, we generated a dataset that matched the sample statistics (*mean* ± *standard error of the mean*, SEM) of naive and trained Canton-S flies from Fig. 1b in Gegear *et al.*[6] (Fig. S1A, see the R script below). Following the procedure from the original paper, we first analyzed our synthetic dataset with the same inappropriate statistical approach: indeed, the one-sample *t*-test resulted in a very low *p*-value of 0.0026 for naive avoidance of the magnetic field and $p < 0.001$ for the magnetic field preference of trained flies (Fig. S1B).

However, when we applied a Generalized Linear Model (GLM) with the appropriate binomial error structure, the effect of the magnetic field was far from significant (ANOVA of GLM: avoidance of naive flies: $p = 0.73$; preference of trained flies: $p = 0.55$; naive vs. trained flies: $p = 0.51$, Fig. S1C). Compared to the *t*-test, where the *SEM* is calculated based on within-

sample variability ($SEM = \delta / \sqrt{N}$) where δ is the standard deviation and N is the sample size), in GLM with the binomial error structure the *SEM* depends on the mean proportion and the sample size ($SEMprop = \sqrt{\hat{P}_M (1 - \hat{P}_M) / N}$ where $\hat{P}_M$ is the mean proportion and N is the sample size, i.e. the number of independent biological replicates). The latter results in wide, largely overlapping confidence intervals for both groups of flies (Fig. S1C).

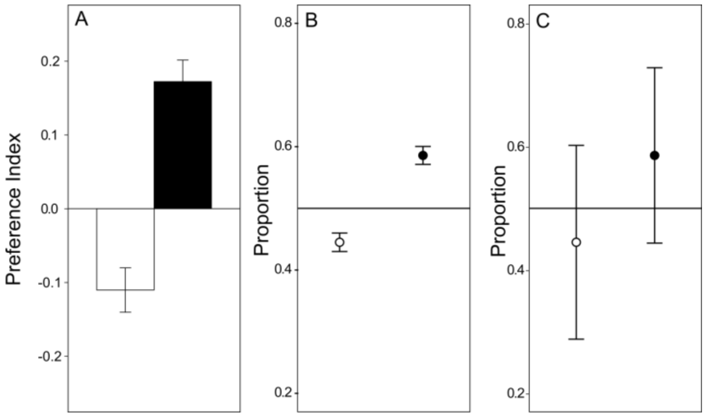

Fig. S1: Treating proportion data as continuous leads to an underestimate of the confidence intervals, which increases the risk of a type I error ("false positive"). We illustrate this by generating a sample of naive and trained flies with the same response to the magnetic field as reported by Gegear *et al.*[6] (Fig. 1b, Canton-S). **A.** Avoidance response by naive (white bar) and preference for the magnetic field by trained flies (black bar) shown as an average Preference Index ± SEM ($\widehat{PI} \pm SEM$; naive flies: –0.11 ± 0.03, trained flies: 0.17 ± 0.03). **B.** The same data presented as a proportion of flies on the magnetic side of the T-maze (mean ± SEM, $\hat{P}_M \pm SEM$; naive flies: 0.445 ± 0.015, trained flies: 0.586 ± 0.015). In Gegear *et al.*[6], the authors neglected the binomial nature of these data, treated proportions as continuous data and hence calculated the *SEM* as: $\delta / \sqrt{N}$. **C.** The same data presented as a proportion of flies on the magnetic side of the T-maze, but with the error bars showing the SEM for proportion data ($SEMprop = \sqrt{\hat{P}_M (1 - \hat{P}_M) / N}$; naive flies: 0.445 ± 0.157, trained flies: 0.586 ± 0.142). Note that the error bars of the groups now largely overlap (i.e., no effect), while the error bars in **B** are shorter by a factor of $\sqrt{100} = 10$ and falsely suggests an effect as a consequence of pseudo-replication, which assumes that each single fly in a set of 100 flies is an independent biological replicate, thereby inflating *N* by a factor of 100.

In fact, the effect size of the magnetic stimulation (Cohen's effect size[2], *h*),

$$h = 2\left(\sin^{-1}\sqrt{\hat{P}_{M1}} - \sin^{-1}\sqrt{\hat{P}_{M2}}\right),$$

reported in Gegear *et al.*[6] is rather small not only for avoidance of naive flies (h = –0.11, $\hat{P}_{M1}$ = 0.445, against a chance probability of $\hat{P}_{M2}$ = 0.5) and preference of trained flies (h =

0.17, $\hat{P}_{M1}$ = 0.5855, against a chance probability of $\hat{P}_{M2}$ = 0.5), but even for the difference between naive and trained flies (h = 0.17 – (–0.11) = 0.28). For the largest magnetic field effect size (h = 0.28), a total sample size N of ca. 400 groups of flies would be required to achieve significance at the p-level = 0.05 and power of 80%. According to our calculations, even if we combine the total number of samples for which Gegear *et al.*[6] reported a magnetic field effect (249 samples), this would still not be enough to achieve significance at the given maximum effect size of h = 0.28.

# Generating the data matching the sample statistics for naive and trained flies from Gegear et al Fig.1b, Canton-S wild-type

```
N1=10; sem1=0.03;  PI1 =–0.11;   R1=(1+PI1)/2   # 0.445 naive, n=10
N2=12; sem2=0.029; PI2 = 0.171; R2=(1+PI2)/2    # 0.5855 trained n=12
ntrials=100 # assumed set size in each experiment
set.seed(48984) # seed for match for "naive" flies
df1=data.frame(succ=rbinom(N1,size=ntrials,R1),setsize=ntrials,cond="naive")
set.seed(12588) # seed for match for "trained" flies
df2<-data.frame(succ=rbinom(N2,size=ntrials,R2),setsize=ntrials,cond="train")
dfr=rbind(df1,df2)
dfr$cond=as.factor(dfr$cond)
dfr$prop = dfr$succ / dfr$setsize # Pm, proportion of flies at the magnetic site of the T-maze
```

# Statistics following the procedure in Gegear et al.

```
t.test(subset(dfr, cond=="naive")$prop,mu=0.5, alternative="less") # avoidance of naive flies, one-sample t-test
t.test(subset(dfr, cond=="train")$prop,mu=0.5, alternative="greater") # preference of trained flies, one-sample t-test
t.test(dfr$prop ~ dfr$cond) # naive vs. trained flies, t-testsummary(aov(dfr$prop ~ dfr$cond)) # naive vs. trained flies, ANOVA
```

# Statistics considering binomial error structure, GLM

```
summary(glm.out<-glm(prop~cond,data=dfr,family=binomial)) # "naive" group is used as a baseline by default, p-level for Intercept corresponds to the one-sample test for "naive" group
dfr$cond = relevel(dfr$cond, ref = "train") # by changing the baseline level we can test the preference of "trained" flies for the magnetic field
```

summary(glm.out<-glm(prop~cond,data=dfr,family=binomial))#now p-level for Intercept corresponds to the one-sample test for "trained" group

anova(glm.out,test="Chisq") # ANOVA of GLM used to calculate p-values for factor "condition", see supplementary results

predict(glm.out, newdata=data.frame(cond=c("naive","train")), type="response",se.fit = TRUE) # calculating the SEMprop

# Computing Cohen's effect size h and required sample size according to power calculations

library(pwr)

ES.h(0.5, 0.455) # Cohen's h for "naive" group

ES.h(0.5858, 0.5)# Cohen's h for "trained" group

ES.h(0.5858, 0.455) # Cohen's h for "naive" vs. "trained"

power.prop.test(p1=0.5855,p2=0.445,power = 0.8, sig.level = 0.05, alternative = "two.sided")

# number n of independent biological replicates per group required to limit false positive ratio to 0.05 and achieve a power of at least 0.8, for the two proportions 0.5855 vs. 0.445; here n=197 per group, i.e. 394 sets of flies in total

pwr.p.test(n = 400, power = 0.8, sig.level = 0.05, alternative = "two.sided")

# the minimum effect size we would be able to reveal with a sample size of 400 when testing a single proportion against chance level of 0.5; here h = 0.14;

# The summary Table S4 (section 1.2.4) shows that we indeed had a total sample size of n=400 when testing naïve flies for spontaneous avoidance of or preference for a magnetic field (two-sided hypothesis)

1.2 Supplementary results

1.2.1 Statistical analysis

Model	Test parameter	*p*-value
glm(P_M ~*line***status*, family=binomial**)**	intercept	0.950
	line	0.900
	status	0.983
	line*status	0.987
lm(*PI*~*line***status*)	intercept	0.666
	line	0.386
	status	0.883
	line*status	0.910

Table S1: Summary of the statistical models fitted to the T-maze data, using the factor variables *line* (levels "Canton-S" (CS-OX) and "*w*;Canton-S" (CS-LE)) and *status* (levels "naive" and "trained"). The interaction term *line***status* is necessary to test for differences in status within a line. The intercept parameter is the common bias over all groups in each model and specifically in the GLM model refers to the parameter $\log\left[P_M/\left(1-P_M\right)\right]$, which is zero for $P_M = 1/2$. All parameters are indistinguishable from zero in both models, which suggests no differences between lines, between status, and between status within lines.

1.2.2 Tests with lower sucrose concentrations

A small number of experiments were done in which *Drosophila* strains we tested at the following lower sucrose concentrations: 0.2, 1.0, 1.5, and 2.0% w/w (Fig. S2 which also includes results for two additional *Drosophila* strains and wild-caught *Drosophila*).

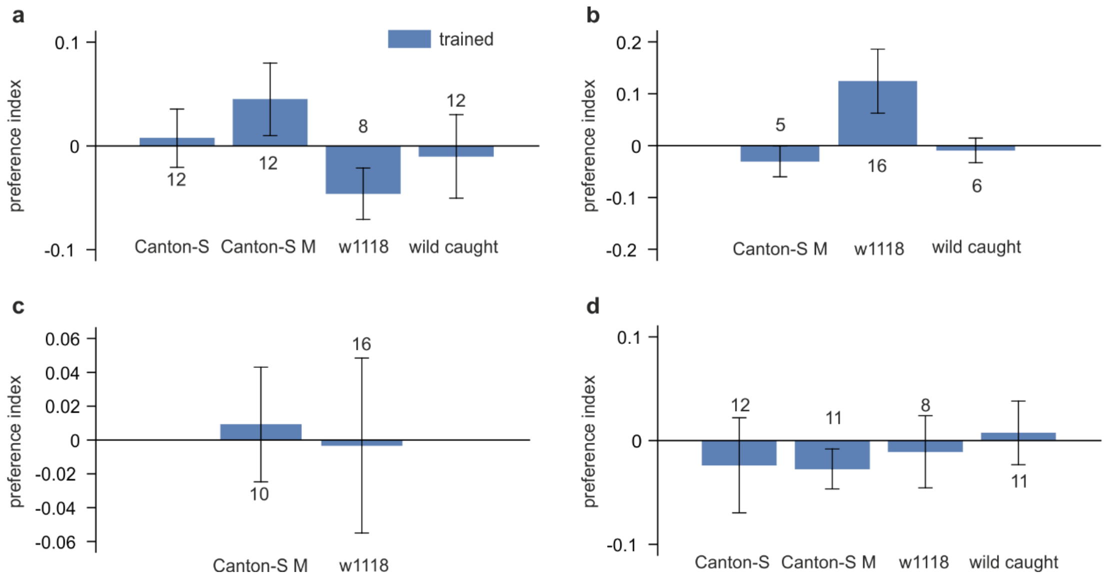

Fig. S2. Flies were trained using lower sucrose concentrations in the reward. **a-d**, 0.2, 1.0, 1.5, and 2.0% w/w, respectively. No magnetic field effect was found. The data are shown as mean *PI* ± *SEM*. The numbers indicate how many groups of flies (of ca. 100 individuals) were tested for each line (Canton-S, Canton-S M, w^{1118}, and wild-caught flies). ANOVA of the GLM is summarised in Table S2. Even though the GLM is calculated on proportions, we decided to present the data as *PI* to be consistent with the original publication[6].

		Df	*Resid. Df*	*p*-value
Fig. S2a	Null		43	
	Stock	3	40	**0.9977**
Fig. S2b	Null		26	
	Stock	2	24	**0.9346**
Fig. S2c	Null		25	
	Stock	1	24	**0.9754**
Fig. S2d	Null		41	
	Stock	3	38	**0.9998**

Table S2. ANOVA of the GLM of the data represented in Fig. S2. The factor "Stock" indicates the *Drosophila* lines used. "*Df*" stands for Degrees of Freedom for a factor variable and indicates the number of factor levels minus 1. "*Residual Df*" stands for Residual Degrees of Freedom and is calculated as the sample size minus 1 (for the Null model) or as the sample size minus 1 minus *Df* (for the factorial model). The *p*-value for the factor variable indicates whether the inclusion of the factor variable yields a significant improvement of a factorial model over the Null model. All *p*-values are much larger than 0.05, hence the factor variables have no significant effect. In conclusion, the preferences observed in our T-maze experiments are indistinguishable from random.

1.2.3 Tests under different conditions

We further tested whether other parameters such as light entrainment, absence of a shielding environment and age of the flies could affect their ability to associate a magnetic field with a sucrose reward. The idea behind these tests was to try to present a more “natural” environment. To do so, we entrained the flies to a natural light:dark photoperiod (instead of a 12:12 cycle) and tested them in a non-electromagnetically shielded environment (Fig. S3a,b). Also, instead of using only ≤ 5-day old flies, we tested groups of flies with a variety of ages (i.e. young and old individuals were pooled together, Fig. S3c).

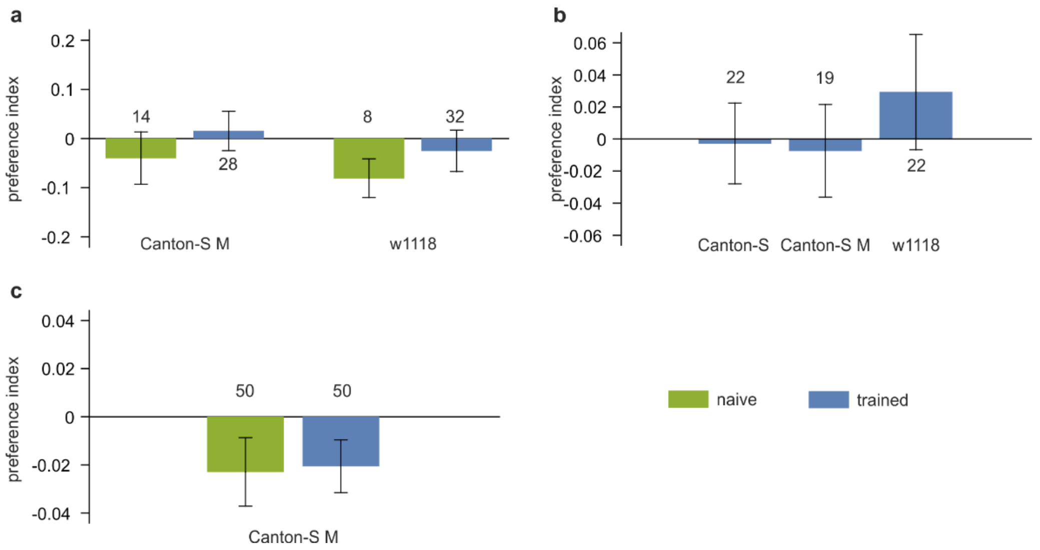

Fig. S3. a, Results for flies tested in “natural” conditions. The flies were reared with a natural light-dark cycle and tested in an electromagnetically unshielded environment. Green and blue bars show naive and trained groups of flies, respectively. **b**. Same conditions as (a) except that green dye had been added to the sucrose solution. Only flies with green abdomens (indicating intake of the coloured solution) were counted as “trained”. **c.** Test for a possible age effect. Flies of all ages were tested. In no case was a magnetic field effect found. The data are shown as mean *PI* ± *SEM*. The numbers indicate how many groups of flies (of ca. 100 individuals) were tested for each line. ANOVA of the GLM is summarised in Table S3. Even though the GLM is calculated on proportions, we decided to present the data as *PI* to be consistent with the original publication[6].

		Df	*Resid. Df*	*p*-value
Fig. S3a	Null		81	
	Status	1	80	**0.8455**
Fig. S3b	Null		125	
	Feeding	1	124	**0.9606**
	Stock	2	122	**0.9773**
Fig. S3c	Null		100	
	Status	1	99	**0.9708**

Table S3. ANOVA of the GLM of the data represented in Fig. S3. The factor "Status" indicates the two possible levels "naive" or "trained". The factor "Stock" indicates the *Drosophila* lines used. The factor "Feeding" indicates whether the flies fed or did not feed on the sucrose reward embedded with green dye. "*Df*" and "*Residual Df*" as in Table S2. The *p*-value for the factor variable indicates whether the inclusion of the factor variable yields a significant improvement of a factorial model over the Null model. All *p*-values are much larger than 0.05, hence the factor variables have no significant effect. In conclusion, the preferences observed in our T-maze experiments are indistinguishable from random.

1.2.4 Summary of T-maze experiments

Experiment	Figure	Genotype	Status	Groups	Flies
Replication	Fig. 1a	Canton-S OX	Trained	100	9,666
			Naive	200	20,669
	Fig. 1b	Canton-S LE	Trained	100	10,674
			Naive	200	21,465
[sucrose] = 0.2%	Fig. S2a	Canton-S	Trained	12	1,275
		Canton-S M	Trained	12	1,103
		w1118	Trained	8	782
		wild caught	Trained	12	1,209
[sucrose] = 1.0%	Fig. S2b	Canton-S M	Trained	5	497
		w1118	Trained	16	1,604
		wild caught	Trained	6	581
[sucrose] = 1.5%	Fig. S2c	Canton-S M	Trained	10	876
		w1118	Trained	16	1,676
[sucrose] = 2.0%	Fig. S2d	Canton-S	Trained	12	1,269
		Canton-S M	Trained	11	962
		w1118	Trained	8	819
		wild caught	Trained	11	1,055
Natural conditions	Fig. S3a	Canton-S M	Trained	42	2,275
		w1118	Trained	40	2,356
Natural conditions + green dye	Fig. S3b	Canton-S	Trained	22	1,889
		Canton-S M	Trained	19	1,463
		w1118	Trained	22	1,376
Age	Fig. S3c	Canton-S	Trained	100	12,117

**Totals**					
			**Trained**	**584**	**55,524**
			**Naive**	**400**	**42,134**
				**984**	**97,658**

Table S4. Summary of the T-maze experiments. The Table gives the name of each experiment, the Figure in which the results are shown, the strains of *Drosophila* used in each experiment, their status (trained or naive), the numbers of groups of flies of ca. 100 individuals tested and the total number of individuals tested.

1.3 Odour conditioning experiments

CS-OX flies were tested for their ability to associate an odour with the sucrose reward employed in the magnetic conditioning experiments (Supplementary Methods). The results are shown in Table S5 and Fig. S4.

**Odorant**	***n***	**mean CS+ ratio**	**SEM**	**1.96 SEM**	***p*-value**
MCH	26	0.662	0.093	0.182	> 0.05
OCT	26	0.654	0.093	0.183	> 0.05
Pooled	52	0.656	0.063	0.126	0.025

Table S5. Results of the positive control experiments, using MCH (4-methylcyclohexanol) and OCT (octan-3-ol) as conditioning stimuli, and using proportion statistics. Columns 3, 4 and 5 contain, respectively, the mean proportion of flies that chose the port in which the conditioned stimulus was presented (CS+ ratio), the standard error of the mean proportion (SEM), and 1.96 SEM. The 95% confidence interval (mean ± 1.96 SEM) for both odours (n = 26) contains the 50% null hypothesis and is therefore only marginally significant. The 95% confidence interval for the pooled sample is [0.53, 0.78], which implies statistical significance at the 5% level (p = 0.025, n = 52).

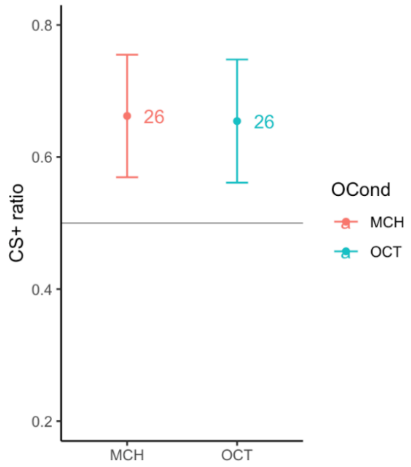

Fig. S4. Results of the positive control experiments, using MCH (4-methylcyclohexanol) and OCT (octan-3-ol) as conditioning stimuli, showing mean ± SEM. n = 26 biologically independent tests for each odorant.

2. Negative geotaxis assay

2.1 Statistical considerations on Fedele *et al.*[9]

Taking advantage of the video recording and tracking of the flies during the trials (Supplementary Methods) we were able to improve the temporal and the spatial resolution of the climbing behaviour. This also allowed us to avoid the unnecessary and artificial categorization of the behaviour (climbers vs. non-climbers) based on somewhat arbitrary criteria (minimum height of 15 cm, maximum time of 15 s).

In the previous work[9], the continuous behavioural response (position of flies) was binarized into two mutually exclusive categories (i.e. flies that climbed more than 15 cm in 15 s vs. flies

that climbed less than 15 cm in 15 s). Importantly, such a procedure requires bimodally distributed responses to produce robust categories. However, when we looked at the distribution of the flies' position in the tubes at the 15 s time point, we observed no clear bimodality either in the control group or when the flies were exposed to a magnetic field (Fig. S5).

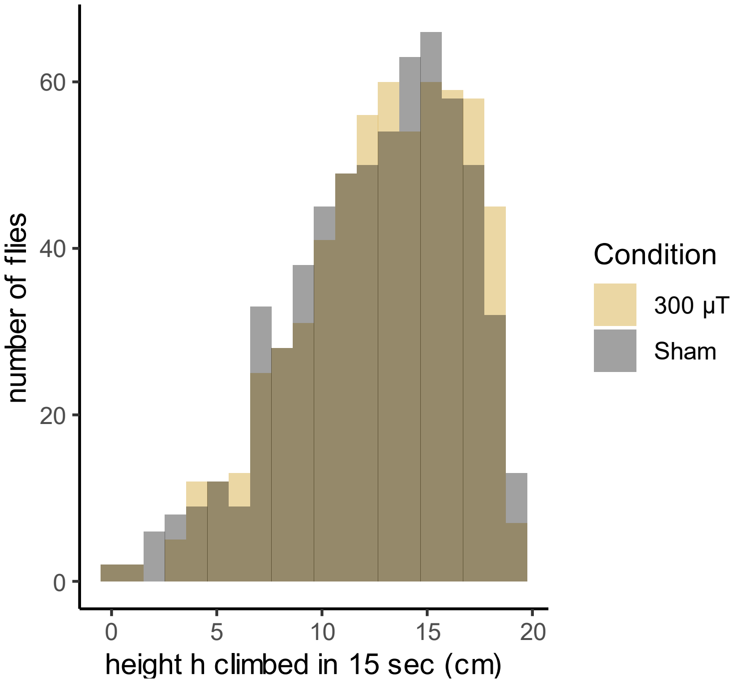

Fig. S5. The distribution of flies in the tubes at the 15 s time point, in the "sham" condition (no applied magnetic field, grey) and in a 300 μT magnetic field (light brown). As clearly visible, the distribution is not bimodal, hence there is no justification for a binary division of the data based on climbers vs non-climbers. The histogram is based on the behaviour of 150 flies per condition (sham and exposure) with 5 repeats.

By treating this type of data as proportional one could easily overestimate the actual difference between responses to different conditions. For example, if ten flies from an exposed group at time point 15 s have climbed (14.6, 15.0, 13.5, 14.5, 16.5, 16.2, 15.4, 14.6, 14.9, and 14.4) cm, while another ten flies from a sham group climbed (15.0, 15.9, 15.3, 13.4, 15.9, 15.3, 13.8, 15.2, 15.1, and 15.1) cm, in the exposed group we would have 30% of the flies that had climbed above 15 cm, while in the sham group it would be 70%. At the same time the average position of the flies in these two groups is virtually the same (mean ± s.d.: 15.0 ± 0.9 vs 15.0 ± 0.8 in the experimental and sham group respectively).

To overcome this problem one could either increase the number of tested flies per tube, thus minimizing the within-group variance or perform the negative geotaxis experiments with a single fly per tube. The latter has several advantages: it excludes any possible group effect and allows one to precisely track and analyse individual climbing behaviour over time.

Having been unable to detect any magnetic field effect on the behaviour of single flies, we estimated the "sensitivity" of our FlyVac set-up, i.e. what was the minimum difference in the climbing behaviour of flies that we would have been able to detect as significant. This was only done the single-fly experiments because this was the most highly optimised set-up. To do so, we modelled the climbing curves for the flies assuming that the magnetic field exposure would reduce the climbing speed of the flies as reported in the original study[9]. For each fly, in every trial of the control condition (blue light, "Sham" exposure, Canton-S LE line; total $N = 208$ flies) we calculated the average climbing speed (v) as the difference between its highest and lowest detected positions (in cm) divided by 30 s (the duration of one trial). The average speed ± s.d. of the control group was $v = 0.396 \pm 0.173$ cm s^{-1}. To model the effect of the magnetic field, we subtracted either 0.5 cm or 1.0 cm from the highest climbing point for each fly and calculated the adjusted climbing speed. This corresponded to a difference in the speed of 0.016 cm s^{-1} (4% of the average speed) and 0.033 cm s^{-1} (8% of the average speed), for 0.5 cm and 1.0 cm, respectively. Using the adjusted climbing speed we then modelled the climbing curve for each fly and compared the control and the modelled groups using the Linear Mixed Effect model (LME). After the 1.0 cm adjustment (but not the 0.5 cm adjustment), the two groups were significantly different from each other (ANOVA of LME: $p = 0.045$). Therefore, if animals showed a robust and continuous response to the magnetic stimuli, we would have been able to detect a difference of ca. 8% in the average climbing speed between sham and exposed groups (Fig. S6).

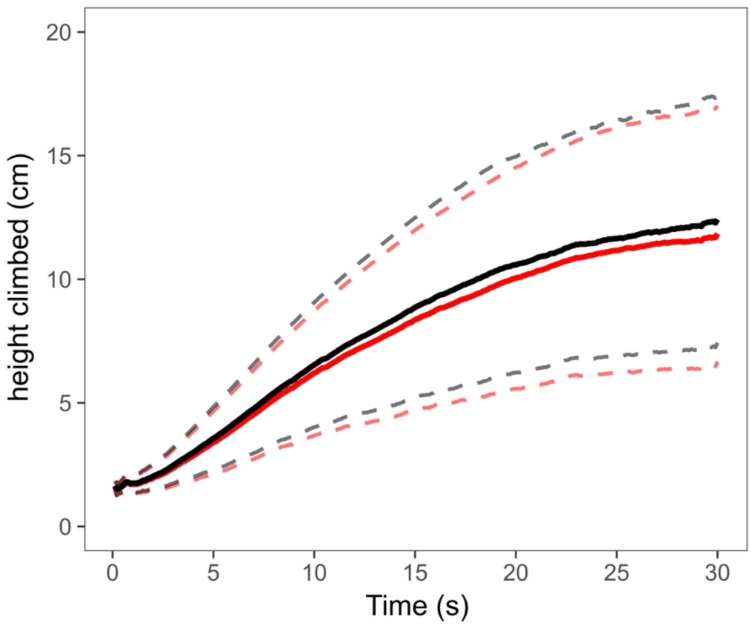

Fig. S6. Minimal detectable difference between groups in the FlyVac set-up. The black line shows the average climbing speed of 208 Canton-S LE flies tested in blue light in the presence of a "sham" stimulus. The red line models the minimum detectable difference if a magnetic field would have reduced the ability of the flies to climb a tube as originally reported[9]. These two groups are significantly different from each other (ANOVA of LME: $p = 0.045$). The actual minimum detectable difference is evaluated as 8% of the average climbing speed. The data are shown as mean (solid lines) ± s.d. (dashed lines).

2.2 Figure 2 climbing statistics

To analyze the percentage *pc* of flies that were able to climb 15 cm in 15 s, we first treated the dependent variable *pc* in the same way as in the original work, i.e. as if it had a Gaussian error distribution, and used a three-way mixed ANOVA, with two between-subject factors with two levels each (light condition, "Blue" vs "Red", and magnetic field exposure, "Exposed" vs "Sham") and one within-subjects factor (10 technical repeats of a given factor combination, in sequences of 5 each, separated by 15 min). We use anova_test in the R-package *rstatix* with the following syntax:

```
anova_test(dataCS_OX,dv=pc,wid=ID,between=c(Light,MF),
     within=c(Repeat,pVid),effect.size = 'pes')
```

where ID is the subject ID variable, i.e. a specific set of flies, tested 10 times in a given factor combination and pVid = 0 and 1 denote the first and second sequence of 5 repeats each. In total, 6 independent testing conditions exist: blue light 500 µT MF = Exposed, blue light 500 µT MF = Sham, blue light 300 µT MF = Exposed, blue light 300 µT MF = Sham, red light 500 µT MF = Exposed, red light 500 µT MF = Exposed. In each of the 6 conditions, 15

independent biological replicates (a set of 10 flies each) were tested, with three independent replicates tested simultaneously in three tubes. In total, we had 89 and 90 evaluable independent replicates for the CS-OX and CS-LE lines, respectively.

CS-OX<br>Effect:	Degrees of freedom, df_1, df_2	F-value	p-value	Partial effect size
Light	1, 85	31.762	$< 10^{-6}$	0.272
MF	1, 85	0.197	0.66	0.002
Repeat	4, 340	14.411	$< 10^{-10}$	0.145
pVid	1, 85	6.558	0.012	0.072
Light:MF	1, 85	0.265	0.61	0.003

Table S6. ANOVA output table for CS-OX. For a single factor, df_1 is the number of factor levels minus 1, and df_2 is the reduced number of independent biological replicates. For the interaction Light:MF, df_1 is the (number of levels in factor Light minus 1) times the (number of levels in factor MF minus 1).

From Table S6 for CS-OX one can see a strong effect of the wavelength of light on the climbing performance, as well as large variability among the technical repeats, and some variability between the two sequences of technical repeats. Magnetic field exposure, both as single factor and in the interaction with light (Light:MF), is insignificant (extremely low effect sizes, if any), so that the null hypothesis of no magnetic effects cannot be refuted.

CS-LE<br>Effect:	Degrees of freedom	F-value	p-value	Partial effect size
Light	1, 86	5.469	0.022	0.06
MF	1, 86	0.002	0.96	0.00002
Repeat	4, 344	16.873	$< 10^{-10}$	0.164
pVid	1, 86	2.050	0.15	0.023
Light:MF	1, 86	0.024	0.88	0.0003

Table S7. ANOVA output table for CS-LS.

In Table S7 for the CS-LS line, one can see that light has a small but significant effect on climbing performance. Again, there is large variability among the technical repeats, and some variability between the two sequences of technical repeats. Magnetic field exposure, both as single factor and in the interaction with light (Light:MF), is insignificant (extremely low effect sizes, if any), so that the null hypothesis of no magnetic effects cannot be refuted.

For comparison, we used a General Linear Mixed Model (GLMM), setting the between-subjects factors Light and MF as fixed effects. As random effects, we used the tube position

and repeats (1 to 5) nested in pVid. The depend variable to be fitted by the model is the number of flies (out of 10 flies in a tube) that successfully climbed 15 cm in 15 s (Table S8).

```
lme4::glmer( cbind(n/10) ~ Light*MF + (1|pVid/Repeat) +
(1|Tube),data = dataCS_OX),family="binomial")
```

CS-OX<br>Effect	Estimate	Std. error	*z*-value	*p*-value
(Intercept)	–0.59	0.04883	–12.134	$< 10^{-16}$
Light Red	–0.21	0.56	–3.768	0.0002
MF 1	–0.02	0.044	–0.452	0.65
Light Red:MF	0.02	0.079	0.285	0.78

CS-LE<br>Effect	Estimate	Std. error	*z*-value	*p*-value
(Intercept)	–1.36	0.081	–16.77	$< 10^{-16}$
Light Red	–0.16	0.073	–2.25	0.025
MF 1	0.014	0.057	0.253	0.8
Light Red:MF	–0.023	0.10	–0.220	0.83

Table S8. Summary of results from GLMM, showing a significant decrease of climbing success under red light, but not effects of the magnetic field exposure.

2.3 Gravity apparatus supplementary results

2.3.1 Canton-S LE Blue light

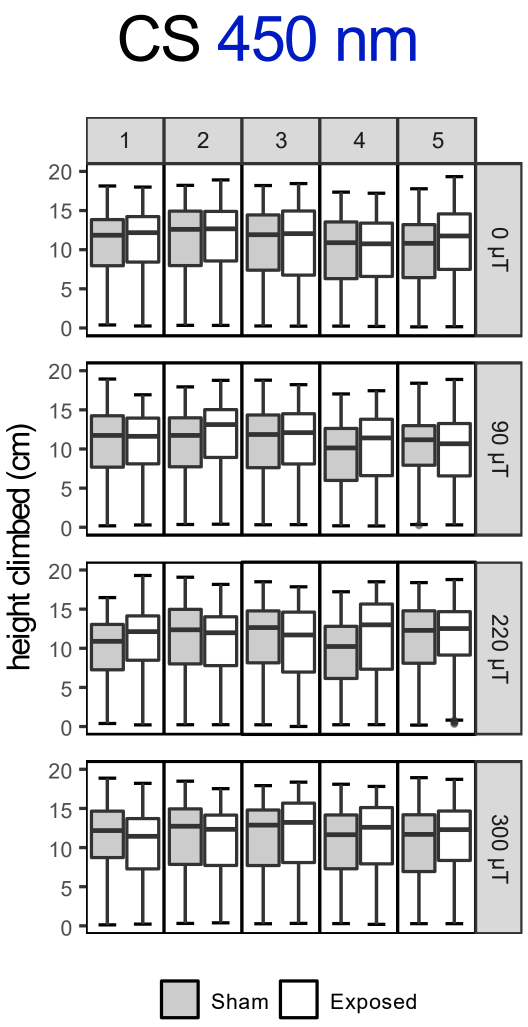

Fig. S7. The data show the average position over time of the flies in three independent tubes over five trials. Flies belonging to the original Canton-S LE line were exposed to different magnetic stimuli (0 µT, 90 µT, 220 µT, 300 µT) in the presence of blue light (410-490 nm). Each treatment (4 magnetic field condition + 4 sham) was tested independently five times giving the total sample size of 15 independent biological replicas per treatment, totalling 120 independent biological replicas. ANOVA output for Linear Mixed Effect model analysis is summarised in Table S9.

2.3.2 Canton-S LE UV-blue light

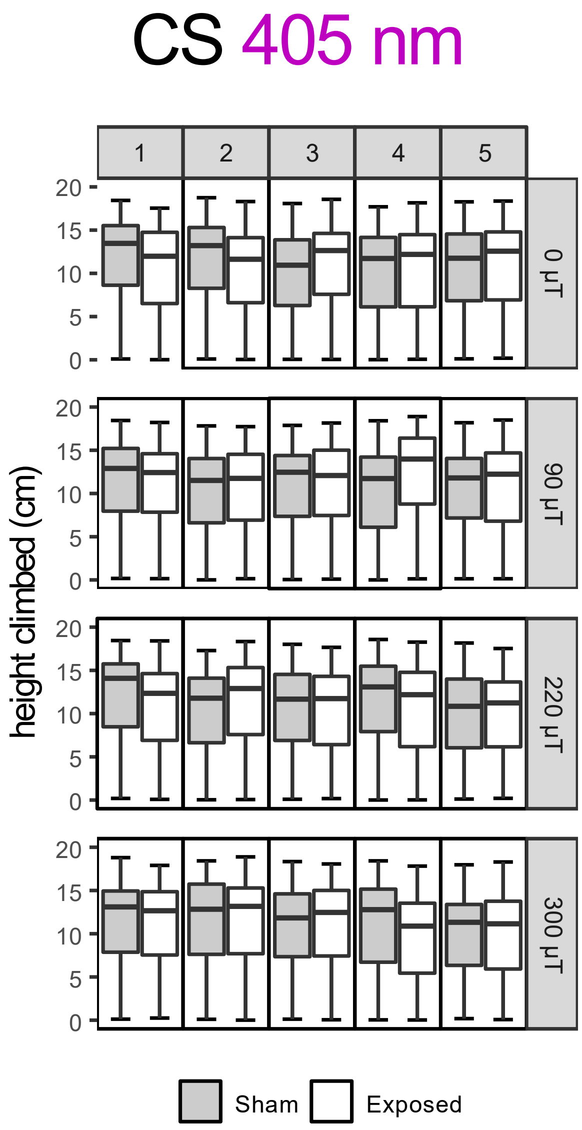

Fig. S8. The data show the average position over time of the flies in three independent tubes over five trials. Flies belonging to the original Canton-S LE line were exposed to different magnetic stimuli (0, 90, 220, 300 µT) in the presence of UV-blue light (380–450 nm).Each treatment (4 magnetic field condition + 4 sham) was tested independently five times giving the total sample size of 15 independent biological replicas per treatment, totalling 120 independent biological replicas. ANOVA output for Linear Mixed Effect model analysis is summarised in Table S9.

2.3.3 Canton-S LE Blue light and radiofrequency exposure

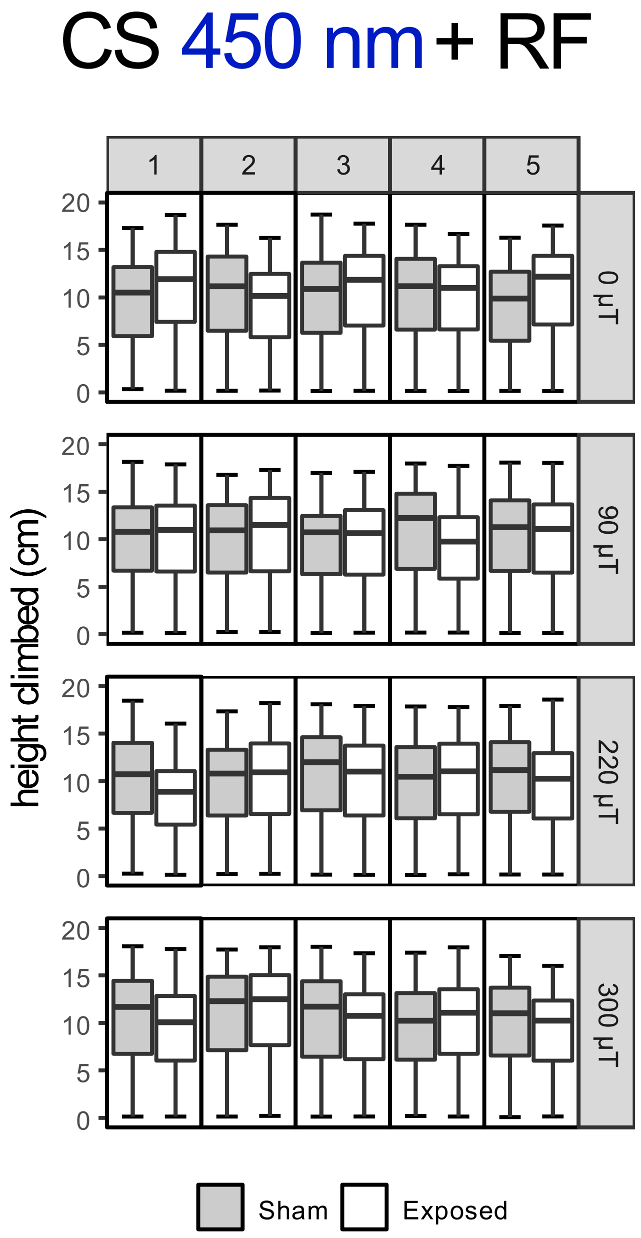

Fig. S9. The data show the average position over time of the flies in three independent tubes over five trials. Flies belonging to the original Canton-S LE line were exposed to different magnetic stimuli (0, 90, 220, 300 µT), in the presence of blue light (410-490 nm) and re-introduced time-dependent magnetic fields. Each treatment (4 magnetic field condition + 4 sham) was tested independently five times giving the total sample size of 15 independent biological replicas per treatment, totalling 120 independent biological replicas. ANOVA output for Linear Mixed Effect model analysis is summarised in Table S9.

2.3.4 Summary

		CS LE 450 nm		CS LE 405 nm		CS LE 450 nm RF	
		F-value	*p*-value	F-value	*p*-value	F-value	*p*-value
Block 1	Exposure	0.0	0.986	7.8	0.013	1.2	0.289
	Condition	0.2	0.924	0.1	0.959	1.6	0.236
	Exposure:Condition	1.1	0.385	1.0	0.424	4.0	0.027
Block 2	Exposure	0.2	0.688	0.0	0.852	0.0	0.950
	Condition	0.4	0.754	1.5	0.247	4.2	0.022
	Exposure:Condition	1.9	0.171	2.2	0.122	1.6	0.221
Block 3	Exposure	0.2	0.703	1.1	0.311	0.5	0.494
	Condition	1.1	0.374	0.4	0.757	1.7	0.213
	Exposure:Condition	0.9	0.482	0.6	0.614	1.6	0.232
Block 4	Exposure	12.8	0.003	0.0	0.906	0.7	0.408
	Condition	5.8	0.007	3.9	0.028	0.1	0.948
	Exposure:Condition	3.0	0.061	8.8	0.001	1.9	0.163
Block 5	Exposure	2.2	0.160	0.2	0.644	0.0	0.914
	Condition	3.1	0.054	2.0	0.152	0.8	0.522
	Exposure:Condition	0.9	0.458	0.1	0.973	2.8	0.075

		F-value	*p*-value	F-value	*p*-value	F-value	*p*-value
All blocks	Exposure	3.7	0.058	0.1	0.807	1.3	0.260
	Condition	1.6	0.196	0.3	0.835	0.3	0.794
	Exposure:Condition	0.3	0.856	1.2	0.301	1.9	0.135

Table S9. Summary of the ANOVA of the Linear Mixed Effect Model of the experiments involving Canton-S LE strain. *p*-values below 0.05 are highlighted in yellow. The upper part of the table is divided into the three main experiments: blue light exposure (450 nm), UV-blue light exposure (405 nm) and blue light and radiofrequency exposure (450 nm RF). Each "Block" corresponds to three biological replicas per magnetic field exposure per magnetic field condition. "Exposure" is the factor describing the presence or absence of a magnetic field stimulus (Sham vs Exposed), while "Condition" is the factor describing the magnetic field intensity (0 µT, 90 µT, 220 µT or 300 µT). In the presence of blue light (450 nm), in one case out of five, there is an Exposure and Condition effect, but there is never an interaction between them. In the presence of UV-blue light (405 nm), in one case out of five, there is an Exposure

effect, a Condition effect and an interaction between the two factors. In the presence of blue light and time-dependent electromagnetic fields (450 nm RF), in one case out of five there is a Condition affect and an interaction between Condition and Exposure, but no Exposure effect. The lower part of the Table summarises all the experimental trials (i.e. considers all five "Blocks" altogether). Importantly, there is no factor (Exposure and Condition) or interaction between them, for which p is less than 0.05. In conclusion, we have found no effect of magnetic field intensity and exposure on *Drosophila* negative geotaxis.

2.3.5 CS- OX Blue light

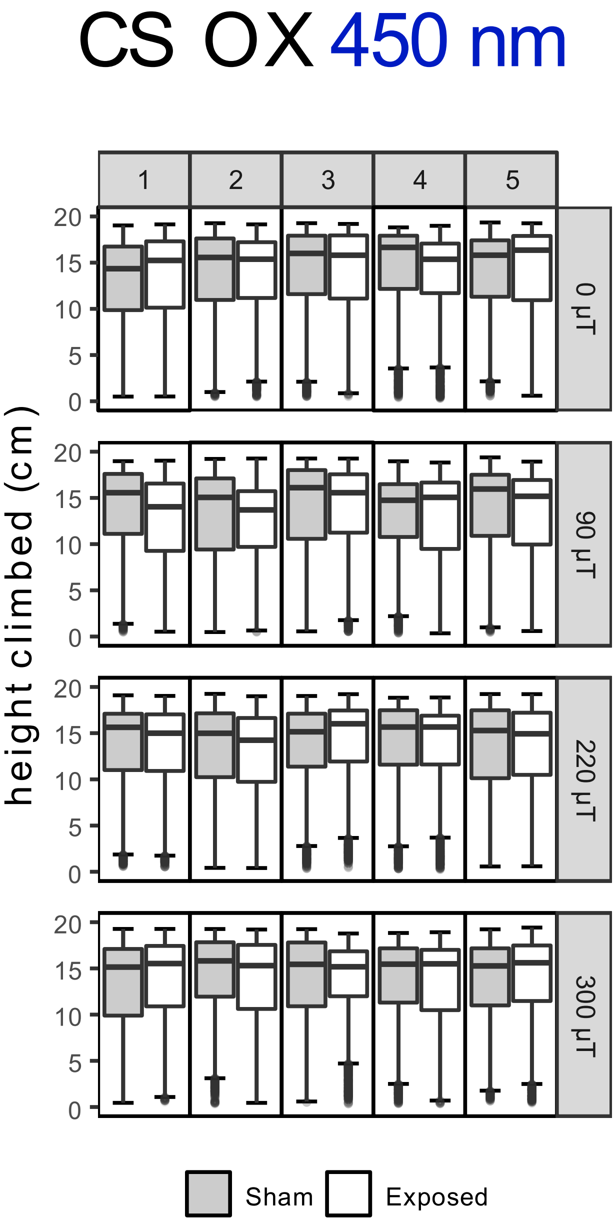

Fig. S10. The data show the average position over time of the flies in three independent tubes over five trials. Flies belonging to the Canton-S OX line were exposed to different magnetic stimuli (0, 90, 220, 300 µT) in presence of blue light (410-490 nm). Each treatment (4 magnetic field condition + 4 sham) was tested independently five times giving the total sample size of 15 independent biological replica per treatment, totalling 120 independent biological replicas. ANOVA output for Linear Mixed Effect Model analysis is summed in Table S10.

2.3.6 CS- OX UV-Blue light

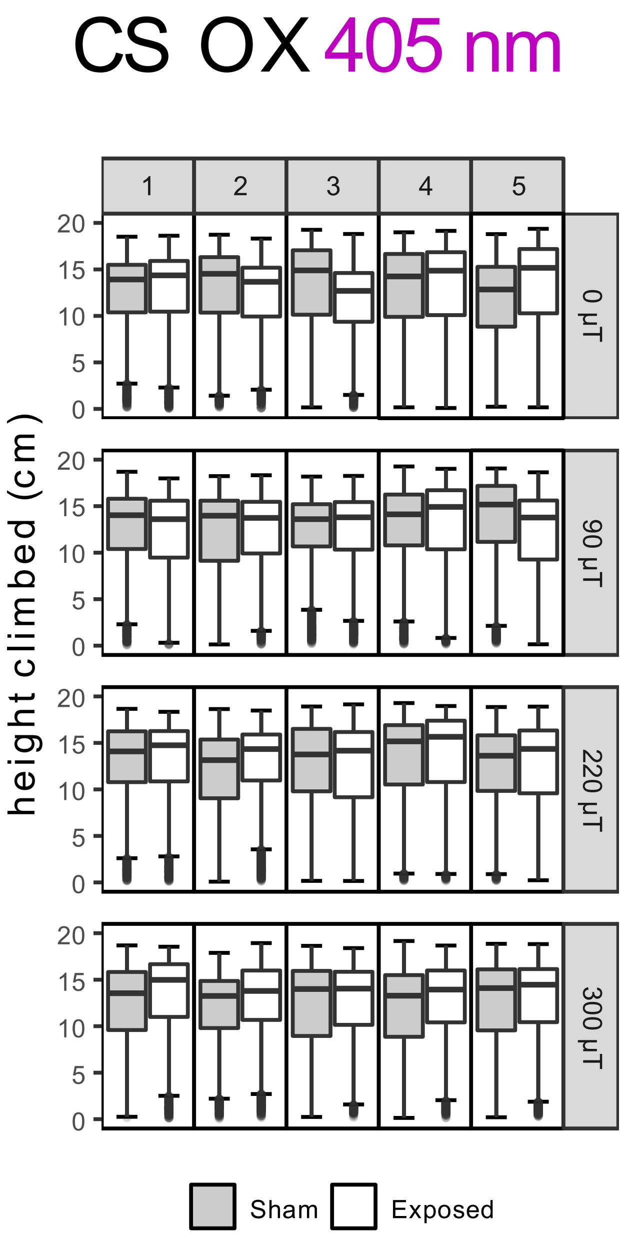

Fig. S11. The data show the average position over time of the flies in three independent tubes over five trials. Flies belonging to the Canton-S OX line were exposed to different magnetic stimuli (0, 90, 220, 300 µT) in presence of UV- blue light (380–450 nm). Each treatment (4 magnetic field condition + 4 sham) was tested independently five times giving the total sample size of 15 independent biological replica per treatment, totalling 120 independent biological replicas. ANOVA output for Linear Mixed Effect Model analysis is summed in Table S10.

2.3.7 CS- OX UV-Blue light and radiofrequency exposure

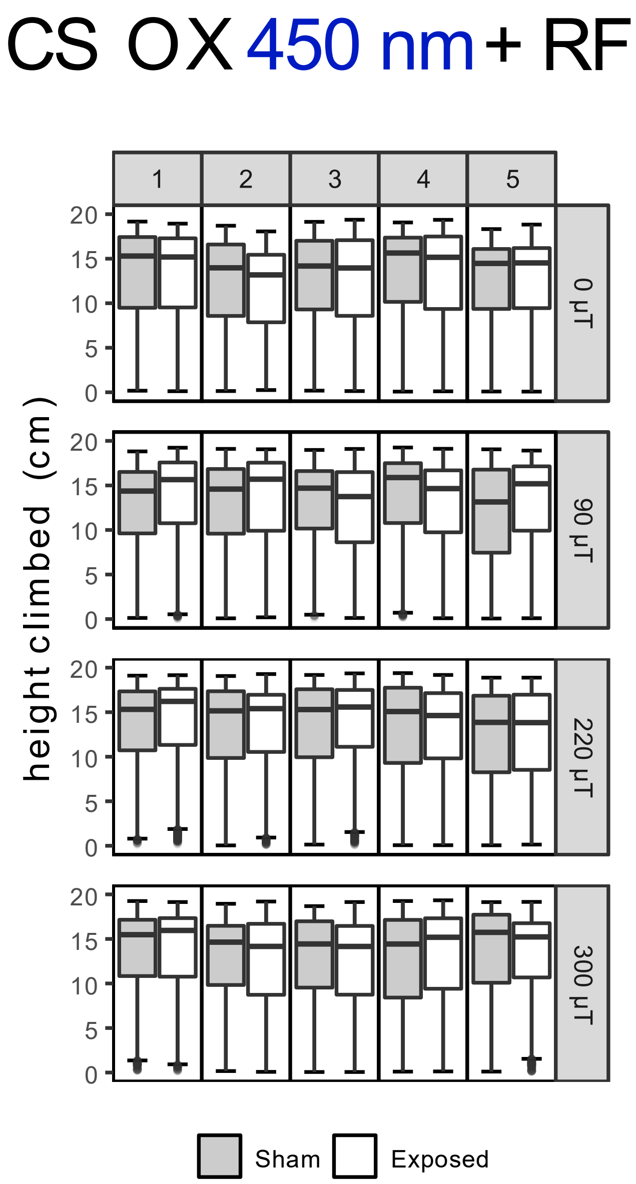

Fig. S12. The data show the average position over time of the flies in three independent tubes over five trials. Flies belonging to the Canton-S OX line were exposed to different magnetic stimuli (0, 90, 220, 300 µT), in presence of blue light (410-490 nm) and re-introduced time-dependent magnetic fields. Each treatment (4 magnetic field condition + 4 sham) was tested independently five times giving the total sample size of 15 independent biological replica per treatment, totalling 120 independent biological replicas. ANOVA output for Linear Mixed Effect Model analysis is summed in Table S10.

2.3.8 Summary

		CS OX 450 nm		CS OX 405 nm		CS OX 450 nm RF	
		F-value	*p*-value	F-value	*p*-value	F-value	*p*-value
Block 1	Exposure	0.0	0.851	1.2	0.282	2.5	0.131
	Condition	0.5	0.704	0.8	0.514	1.3	0.301
	Exposure:Condition	1.9	0.163	1.5	0.263	0.8	0.505
Block 2	Exposure	3.1	0.099	1.0	0.336	0.1	0.742
	Condition	4.7	0.016	0.1	0.952	3.2	0.051
	Exposure:Condition	0.5	0.684	1.8	0.189	0.9	0.485
Block 3	Exposure	0.3	0.581	1.4	0.248	1.0	0.339
	Condition	0.1	0.936	0.2	0.920	3.3	0.048
	Exposure:Condition	0.6	0.618	2.3	0.119	0.7	0.567
Block 4	Exposure	1.3	0.274	2.3	0.147	0.6	0.440
	Condition	2.8	0.076	5.7	0.008	0.4	0.722
	Exposure:Condition	0.3	0.804	0.2	0.877	0.6	0.630
Block 5	Exposure	0.3	0.618	0.8	0.397	2.5	0.135
	Condition	1.3	0.311	0.3	0.851	4.8	0.014
	Exposure:Condition	1.3	0.314	5.6	0.008	2.9	0.069

		F-value	*p*-value	F-value	*p*-value	F-value	*p* -value
All blocks	Exposure	3.3	0.072	1.4	0.234	0.0	0.902
	Condition	3.3	0.022	1.0	0.411	2.2	0.094
	Exposure:Condition	1.0	0.405	2.4	0.070	0.8	0.518

Table S10. Summary of the ANOVA of the Linear Mixed Effect model of the experiments involving Canton-S OX strain. *p*-values below 0.05 are highlighted in yellow. The upper part of the table is divided into the three main experiments: blue light exposure (450 nm), UV-blue light exposure (405 nm) and blue light and radiofrequency exposure (450 nm RF). Each "Block" corresponds to three biological replica per magnetic field exposure per magnetic field condition. "Exposure" is the factor describing the presence or absence of a magnetic field stimulus (Sham vs Exposed), while "Condition" is the factor describing the magnetic field intensities (0 µT, 90 µT, 220 µT or 300 µT). In the presence of blue light (450 nm), in one case out of five, there is a Condition effect, but there is never an Exposure effect or an interaction between them. In presence of UV-blue light (405 nm), in one case out of five, there is a

Condition effect, an interaction between Exposure and Condition but no Exposure effect. In the presence of blue light and time-dependent electromagnetic fields (450 nm RF), in two cases out of five there is a Condition affect, but there is no Exposure effect and no interaction between Condition and Exposure. The lower part of the Table summarises all the experimental trials (i.e. considers all five “Blocks” altogether). In the presence of blue light (450 nm) there is a Condition effect, but no Exposure effect no interaction between them. In the other two experiments, there is no factor (Exposure and Condition) or interaction between them, for which values are below the *p*-level of 0.05. In conclusion, we have found no effect of magnetic field intensity or exposure on *Drosophila* negative geotaxis.

2.4 FlyVac apparatus supplementary results

2.4.1 Canton-S LE UV-blue & blue light

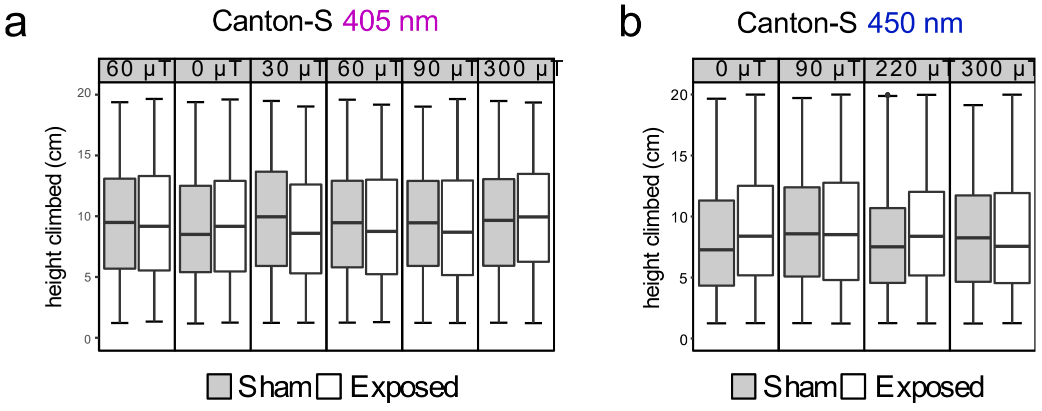

Fig. S13. **a**. Average position of the flies over time over trials of a total of 617 individual Canton-S LE flies when exposed to UV-blue light (380-450 nm) and to different magnetic field intensities. The different magnetic field intensities applied were 60 µT *, 0 µT, 30 µT, 60 µT, 90 µT, 300 µT. Grey boxplots show a sham exposure, white a magnetic field exposure. Each boxplot represents the behaviour of ca. 50 individual *Drosophila*. No magnetic field effect was noticed. ANOVA of the Linear Mixed Effect Model, $p = 0.3944$. **b**. Average position of the flies over time over trials of a total of 411 individual Canton-S LE flies when exposed to blue light (410-490 nm) and to different magnetic field intensities. The different magnetic field intensities applied were 0, 90, 220 and 300 µT. Grey boxplots show a sham exposure, white a magnetic field exposure. Each boxplot represents the behaviour of ca. 50 individual *Drosophila*. No magnetic field effect was noticed. ANOVA of the Linear Mixed Effect Model, $p = 0.4905$. For details see Table S11.

2.4.2 Canton-S LE Red light & pdf^{01} blue light

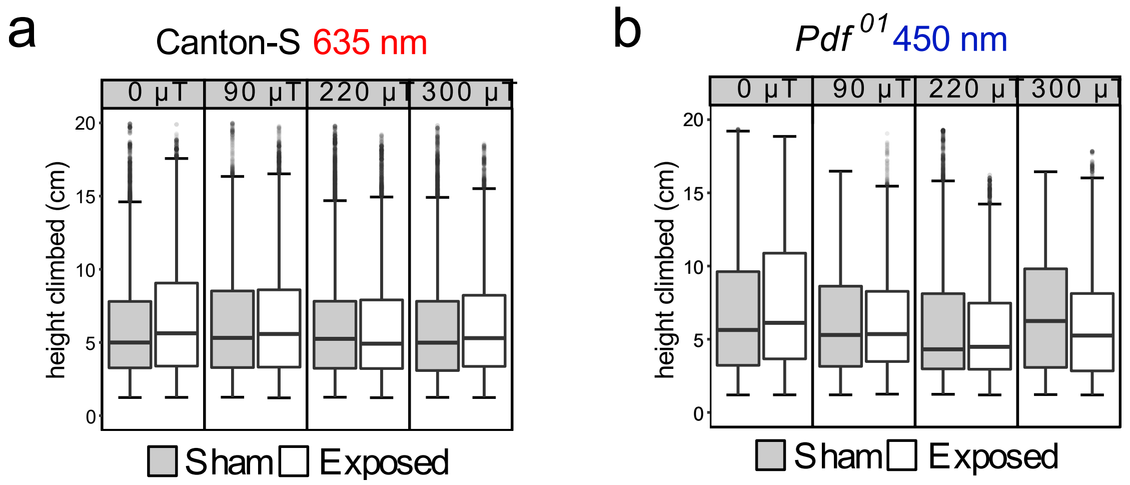

Fig. S14. **a**. Average position of the flies over time over trials of a total of 412 individual Canton-S LE flies when exposed to red light (580–660 nm) and to different magnetic field intensities. The different magnetic field intensities applied were 0, 90, 220, 300 µT. Grey boxplots show a sham exposure, white a magnetic field exposure. Each boxplot represents the behaviour of ca. 50 individual *Drosophila*. No magnetic field effect was noticed. ANOVA of the Linear Mixed Effect Model, p = 0.6855. **b**. Average position of the flies over time over trials of a total of 165 individual *pdf* ^{01} flies when exposed to blue light (410-490 nm) and to different magnetic field intensities. The different magnetic field intensities applied were 0 µT, 90 µT, 220 µT and 300 µT. Grey boxplots show a sham exposure, white – a magnetic field exposure. Each boxplot represents the behaviour of ca. 20 individual *Drosophila*. No magnetic field effect was noticed. ANOVA of the Linear Mixed Effect Model, p = 0.5394. For details see Table S11.

2.4.3 Canton-S OX Blue light

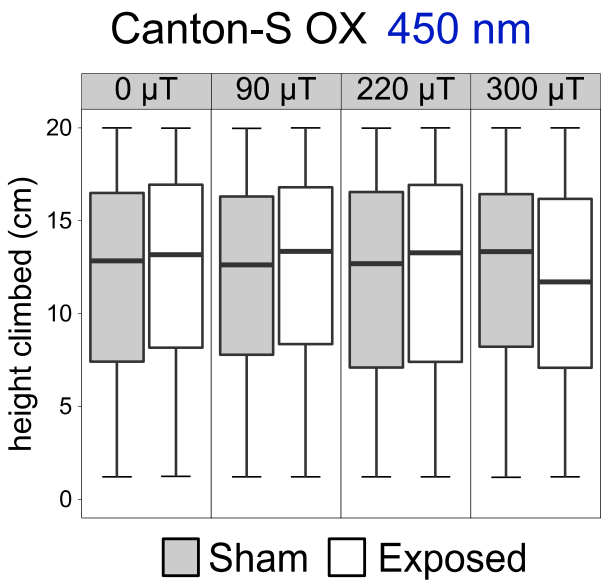

Fig. S15. Average position of the flies over time over trials of a total of 355 individual Canton-S OX flies when exposed to blue light (410-490 nm) and to different magnetic field intensities. The different magnetic field intensities applied were 0 µT, 90 µT, 220 µT and 300 µT. Grey boxplots show a sham exposure, white a magnetic field exposure. Each boxplot represents the behaviour of ca. 50 individual *Drosophila*. No magnetic field effect was noticed. ANOVA of the Linear Mixed Effect Model, $p = 0.1494$. For details see Table S11.

2.4.4 Summary

	Exposure	Condition (µT)	N flies	ANOVA of the LME		
CS LE 405 nm	Exposed	60*	48		F-value	*p*-value
	Exposed	0	51	Exposure	0.0592	0.8079
	Exposed	30	50	Condition	1.689	0.1864
	Exposed	60	55	Exposure:Condition	0.9332	0.3944
	Exposed	90	54			
	Exposed	300	51			
	Sham	60*	55			
	Sham	0	49			
	Sham	30	52			
	Sham	60	51			
	Sham	90	52			
	Sham	300	49			
CS LE 450 nm	Exposed	0	51		F-value	*p*-value
	Exposed	90	49	Exposure	2.2262	0.1365
	Exposed	220	54	Condition	0.9727	0.4055
	Exposed	300	49	Exposure:Condition	0.807	0.4905
	Sham	0	51			
	Sham	90	51			
	Sham	220	53			
	Sham	300	53			
CS LE 635 nm	Exposed	0	56		F-value	*p*-value
	Exposed	90	53	Exposure	0.5002	0.4798
	Exposed	220	55	Condition	0.4193	0.7393
	Exposed	300	49	Exposure:Condition	0.4956	0.6855
	Sham	0	49			
	Sham	90	50			
	Sham	220	48			
	Sham	300	52			
pdf^{01} 450 nm	Exposed	0	18		F-value	*p*-value
	Exposed	90	19	Exposure	0.2145	0.6439
	Exposed	220	20	Condition	1.4854	0.2206
	Exposed	300	26	Exposure:Condition	0.7235	0.5394
	Sham	0	19			
	Sham	90	20			
	Sham	220	24			
	Sham	300	19			
CS OX 450 nm	Exposed	0	41		F-value	*p*-value
	Exposed	90	49	Exposure	0.3813	0.5373
	Exposed	220	40	Condition	0.4027	0.7511
	Exposed	300	43	Exposure:Condition	1.7866	0.1494
	Sham	0	44			
	Sham	90	46			
	Sham	220	44			
	Sham	300	48			

	Total N	1960

*The magnetic field was applied on the z axis but with the direction inverted (the field was pointing upwards)

Table S11. Summary of all the FlyVac experiments. The Exposure factor indicates the presence or the absence of an external magnetic field (Sham vs Exposed), the Conditions factor indicates the intensity of the field to which the flies were exposed. After testing a total of 1960 single flies, independent of the light wavelength, the field intensity and the genotype of the flies tested, there is no factor, or interaction between them, that shows a *p*-value below the 0.05 *p*-level. In conclusion, *Drosophila* negative geotaxis is not disrupted by an external magnetic field.

Supplementary References

1 Baldi, B. & Moore, D. S. *The practice of statistics in the life sciences*. (W. H. Freeman and Company, 2012).

2 Cohen, J. *Statistical power analysis for the behavioral sciences*. (L. Erlbaum Associates, 2008).
